# Map Task Corpus of Heritage BCMS spoken by second-generation speakers in Switzerland

**DOI:** 10.1007/s10579-023-09634-7

**Published:** 2023-02-22

**Authors:** Dolores Lemmenmeier-Batinić, Josip Batinić, Anastasia Escher

**Affiliations:** 1 Slavisches Seminar, University of Zurich, Zurich, Switzerland; 2 Department of Literature, University of Antwerp, Antwerp, Belgium; 3 NEXUS Clinical Bioinformatics, ETH Zurich, Zurich, Switzerland

**Keywords:** Interactive corpus platform, Spoken language, Heritage speakers, Bosnian/Croatian/Montenegrin/Serbian (BCMS)

## Abstract

In this paper, we present a corpus for heritage Bosnian/Croatian/Montenegrin/Serbian (BCMS) spoken in German-speaking Switzerland. The corpus consists of elicited conversations between 29 second-generation speakers originating from different regions of former Yugoslavia. In total, the corpus contains 30 turn-aligned transcripts with an average length of 6 min. It is enriched with extensive speakers’ metadata, annotations, and pre-calculated corpus counts. The corpus can be accessed through an interactive corpus platform that allows for browsing, querying, and filtering, but also for creating and sharing custom annotations. Principal user groups we address with this corpus are researchers of heritage BCMS, as well as students and teachers of BCMS living in diaspora. In addition to introducing the corpus platform and the workflows we adopted to create it, we also present a case study on BCMS spoken by a pair of siblings who participated in the map task, and discuss advantages and challenges of using this corpus platform for linguistic research.

## Introduction

Bosnian/Croatian/Montenegrin/Serbian (BCMS) is one of the most widespread *heritage languages*^1^ in Switzerland. At least 2.4% of the Swiss population speaks BCMS on a daily basis, which corresponds to 173,546 individuals.^2^ However, the BCMS spoken in Switzerland is still under-investigated, despite the increasing body of research on heritage languages (Polinsky & Scontras, 2020) and on heritage BCMS spoken in other German-speaking countries (Hansen, 2018; Hansen et al., 2013; Raecke, 2007; Romić, 2016; Schlund, 2006; Simonović & Arsenijević, 2020). One of the main challenges in the research on heritage varieties is the difficulty in obtaining and redistributing authentic data, which often limits the research settings to case studies based on online communication between peers (Kajgo, 2020; Zagoricnik, 2014). What is particularly lacking for facilitating the research on (BCMS) heritage varieties are corpora of spoken language, since heritage speakers use their heritage language predominantly in oral communication. In order to provide a resource which would enhance the study of heritage BCMS, we created a map task corpus of this variety spoken by second-generation speakers in Switzerland.

The corpus consists of data that have been collected by students during the courses “Corpus linguistics” and “BCMS as heritage language in Switzerland” at the Department of Slavonic Languages and Literatures at the University of Zurich in 2019 and 2020. The aim of the exercises was to train the students to conduct an empirical study on spoken language. The task included fieldwork, transcription of speech, linguistic annotation and analysis of selected phenomena found in the collected data. At the end of the courses, we obtained 30 short transcripts and recordings of BCMS heritage speakers having parents born in former Yugoslavia. In order to present this data to a broader audience, and to present a prototype for a corpus access of this type of spoken data, we created an interactive platform on which users can preselect the transcripts according to particular metadata and frequency distribution of pre-annotated features, sort and filter corpus counts, search the annotated turn-aligned transcripts, and add and export their own custom annotations. Hence, in addition to providing a first publicly available resource for heritage BCMS in German-speaking diaspora, with this corpus, we also present an example for visualising, structuring, and accessing spoken language data. With the corpus architecture used for this prototype, we address the need of user-group specific differentiation when accessing spoken language corpora (Fandrych et al., 2016; Goldman et al., 2005). The map task corpus of heritage BCMS is primarily tailored for linguists investigating heritage BCMS in interaction, and teachers and learners of BCMS living abroad.

### About the label BCMS

The migration from the countries of former Yugoslavia to Switzerland started in the 1960s, when the name of the official language was Serbo-Croatian. However, Serbo-Croatian as standard language has never been entirely uniform: there was an *Eastern* and a *Western* variety, as well as two other (sub-) varieties: *Bosnian–Herzegovinian standard-language expression* and *Montenegrin standard-language expression* (Bugarski & Hawkesworth, 2004). Following the break-up of Yugoslavia, new standard languages have been codified from what was former known as Serbo-Croatian: Bosnian, Croatian, Serbian and Montenegrin. All these standard languages are almost completely mutually understandable, since they are all based on the Štokavian dialect, which is spoken in all of these countries.^3^ While the denomination BCMS is widespread in the linguistic community, Bosnian, Serbian, Croatian and Montenegrin are also seen as separate languages, depending on the definition and approach one adopts when dealing with them. There is an extended body of literature about the controversy of BCMS and whether and under which aspects it can be considered one language (see Langston & Peti-Stantić, 2003; Gröschel, 2009; Bugarski, 2000; Kapović, 2010; Kordić, 2010). For reasons of consistency, in this paper we use the term BCMS to denominate these language(s), although the variety from Montenegro is not included in our data.

### About map task corpora

Map tasks (Anderson et al., 1991; Thompson & Bader, 1993) are elicited conversations used as material for different research questions in linguistic research. In a typical map task setting, two participants take part in a cooperative problem-solving task. Both participants obtain a sheet of paper populated with various images, but only one of them also has a path drawn around the images. The participant who sees a path has the assignment of explaining to their interlocutor how to draw it on their sheet. Depending on the research design, the roles can also be switched, provided that there is a second set of pictures. The first publicly available map task corpus (HCRC) was designed to “furnish a common set of materials for the simultaneous study of several different linguistic phenomena” (Anderson et al., 1991, p. 353). Since then, several map task corpora were created for different research purposes and for different languages. Some of these are Chiba University Japanese Map Task Dialogue Corpus (2007),^4^ Hamburg Map Task Corpus (HAMATAC^5^; Schmidt et al., 2010) and Berlin Map Task Corpus (BeMaTaC^6^; Sauer & Lüdeling, 2016) for German, Montclair Map Task Corpus^7^ (Pardo et al., 2018) for English, and Aix Map Task corpus (Gorisch et al., 2014) for French.

We decided to use map tasks for our pilot corpus of heritage BCMS because map task conversations are thematically similar and hence comparable, and at the same time they represent a source of semi-spontaneous speech that is differentiated enough to allow for the investigation of various speech phenomena. They are well suited for making a preliminary assessment of lexical, phonetic, morphosyntactic and disfluency patterns of heritage speakers, which was our aim in the course “BCMS as heritage language”.

## Map Task Corpus of Heritage BCMS

In this section, we present the idea behind our map task design, the steps we undertook in order to collect the corpus data and a brief description of the participants and the language they used in map tasks conversations.

### Map task design

**Fig. 1:**
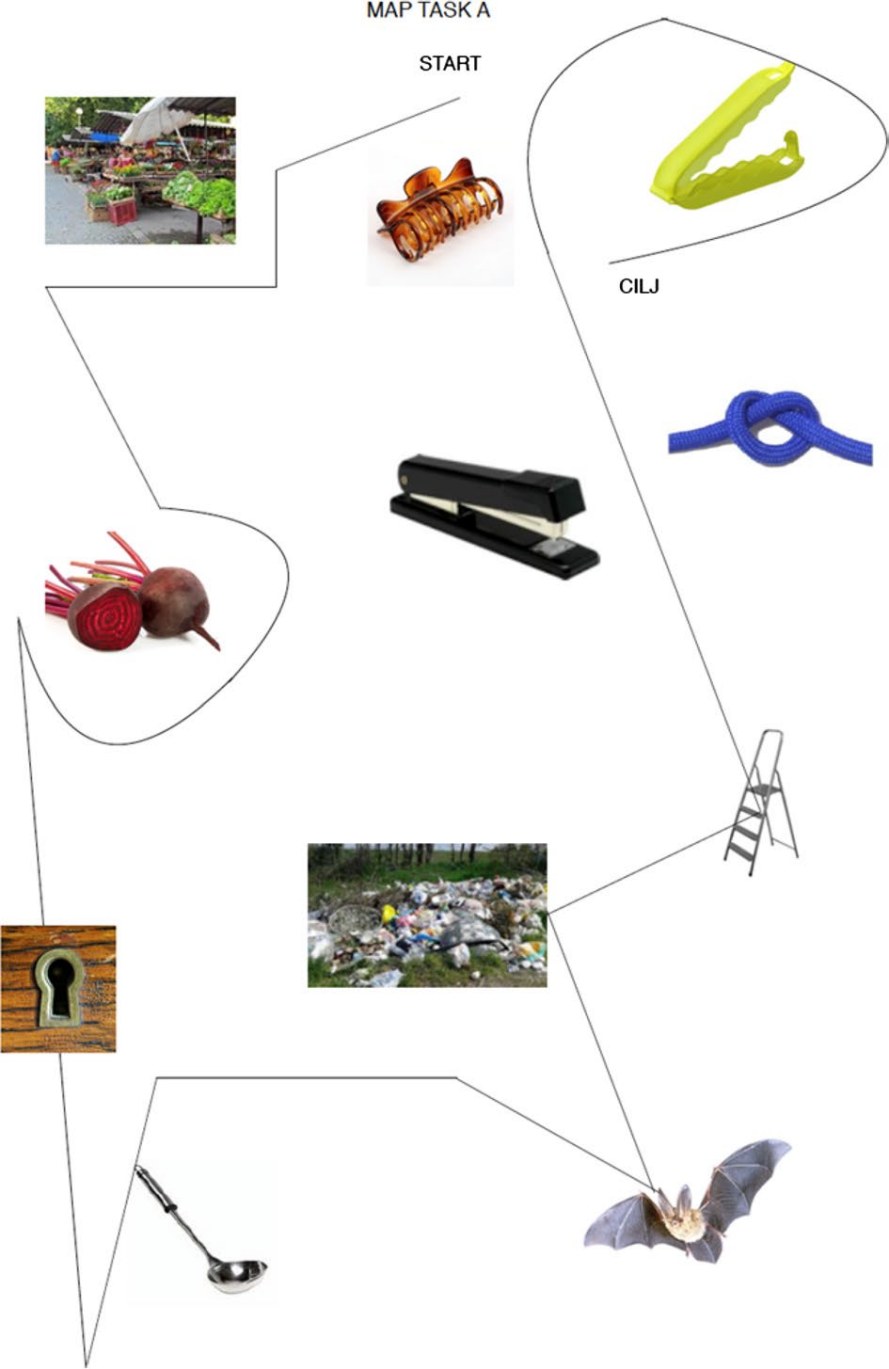
Map tasks used in the experiments (map task A). Market: Gmihail at Serbian Wikipedia, CC BY-SA 3.0 RS https://creativecommons.org/licenses/by-sa/3.0/rs/deed.en, via Wikimedia Commons; bag clip: Rotho; beetroot: kindPNG.com; hair clipper: A&A Hair Beauty; stapler: Ofrex; node: dreamstime.com Pancaketom); key hole: Thegreenj, CC BY-SA 3.0 http://creativecommons.org/licenses/by-sa/3.0/, via Wikimedia Commons; bat: PD-USGov, exact author unknown, Public domain, via Wikimedia Commons: ladder, garbage, soup spoon: unknown author

**Fig. 2:**
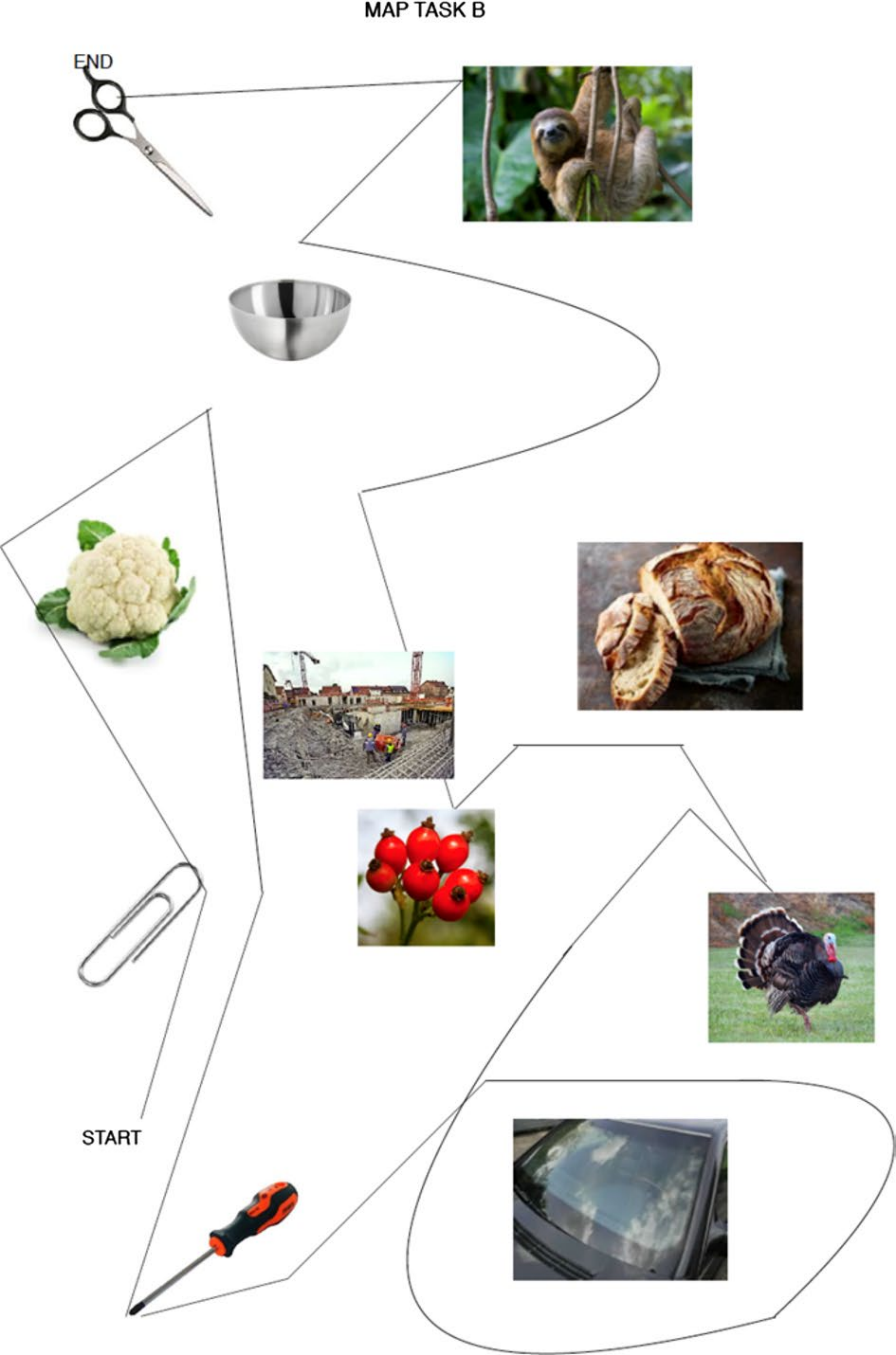
Map tasks used in the experiments (map task B). Scissors: makeup.sk; sloth: Roy Toft; bowl: IKEA; cauliflower: shutterstock (Egor Rodynchenko); construction site: factumArchive; bread: Hubertus Schüler; rose hip: RGBStock.com (micromoth); turkey: dreamstime.com (Mike Neale); screwdriver: Narex; paperclip, windshield: unknown author

We used an adapted version of the original HCRC map tasks (Anderson et al., 1991) with our own set of images. For each map task, we selected 11 images that represent objects/places from everyday life (e.g.: ‘bread’, ‘market’, ‘bowl’), but also rarely used words that we evaluated as challenging for heritage speakers (e.g.: ‘sloth’, ‘paper-clip’) see Figs. 1 and 2.^8^ In selecting the images, we aimed to find terms that differ across BCMS standard varieties (e.g. *kruh/hleb/hljeb* for ‘bread’ in Croatian/Serbian/Bosnian). This was performed in order to be able to assess active and passive knowledge of “own” and “foreign” BCMS varieties in the course “BCMS as heritage language”. Our maps differ from the map tasks presented in the original HCRC Map Task Corpus (Anderson et al., 1991) in that (1) the features on the maps are not labeled, (2) the maps contain mostly features not to be encountered in a real navigation task, and (3) that the giver and the follower had the maps with the same exact features, while they had some differences in HCRC map tasks’ design.

Before conducting the map tasks, we checked that (non-heritage) BCMS speakers (4 test individuals) were familiar with the represented images at least in their BCMS variety. We also checked whether the images were known to Swiss–German native speakers without migration background (5 test individuals). Interestingly, in contrast to BCMS test individuals, Swiss–German speakers were not familiar with the word for *rose hip* and only one of them knew Swiss–German word for *beetroot*. Despite that, we decided to keep these two images in the map tasks because BCMS test persons were familiar with them.

### Data collection

The students’ task was to find at least two BCMS second-generation speakers originating from different regions of former Yugoslavia and to let them engage in map tasks. We defined second-generation heritage speakers as speakers of BCMS who were either born in Switzerland or who have migrated to Switzerland before starting primary school, and whose parents grew up in former Yugoslavia. Before the experiment, participants were asked to sign a declaration of consent to record and use their data for research purposes.^9^ The initial plan was to record the map task conversations in a quiet room with high-resolution audio quality. However, due to COVID-19 restrictions, some map tasks were conducted online and recorded using video conferencing platforms. After each completed task, participants were requested to fill a questionnaire containing questions on the map task, as well as on their language use, social environment, self-assessment and language attitudes toward BCMS. We collected 30 map tasks conversations (overall 12,988 tokens).^10^

### Participants

A total of 29 participants (18 female and 11 male) took part in the task.^11^ The median value for participants’ age was 23 years. Their highest educational attainment was university or higher education diploma (16) and high school (13). On average, the participants assessed their proficiency in BCMS as 4.7 on a scale from 1 to 6 (n = 19^12^). 12 Participants attended classes in BCMS, and 16 did not (n = 28). Most of the participants originated from Bosnia and Herzegovina: there were 11 participants with both parents, and 6 participants with one parent from Bosnia and Herzegovina, and another from other successor states. A total of five participants had both parents originating from Serbia, four had both parents from Croatia, and two had both parents from Kosovo.^13^ The majority of participants were ethnic Serbs (14) and Croats (9).^14^

All participants lived in German-speaking Switzerland at the time of recording. The participants were given a pseudonym for further processing, as well as a “speaker-id”, which refers to the unique identifier for a speaker in a particular map task event.

### Language in the map tasks

When asked about their first language, most participants (including those from Bosnia and Herzegovina) responded Croatian (10) or Serbian (9), and only one specified Bosnian. Other speakers either did not reply (5) or they indicated Serbo-Croatian (1), German (2) and Swiss–German (1) as their first language. While most participants named the language according to their ethnic backgrounds, naming the language proved to be a delicate subject for some participants.^15^ All participants agreed that they would call BCMS *naš jezik* (‘our language’) when they would speak about it with their map task interlocutor. However, when asked if they spoke the same language as their interlocutor, the opinions were divided: out of 19 participants who named language differently than their interlocutor, only 8 (at least partially) agreed with the statement that it is the same language. Nevertheless, all participants spoke the Štokavian dialect in the map task, which is the dialectal basis of all standard BCMS languages. Most of them used Ijekavian (17) and Ekavian (9) variety, while three participants reported to speak Ikavian.^16^

## Corpus compilation

In this section we describe the processes of transcription, normalisation, and creation of the annotated TEI transcripts. Since the recordings contain many non-standard terms, we adopted the solution of transcribing them as they are pronounced, and normalising them in a second step.

### Transcription

The corpus has been transcribed by 11 students with the help of FOLKER^17^ transcribing tool using cGAT conventions (Schmidt et al., 2015). Each student transcribed the map tasks they have previously recorded (which ranges from 2 to 8). Transcribers were given the instructions to transcribe the conversations as they are pronounced, and to keep dialectal features and other non-standard features instead of correcting them to the standard language. Student’s transcripts were reviewed by two tutors. No measurements of inter-transcriber reliability were performed. As proposed by cGAT, we used pronunciation-based (semi-orthographic) transcription, and we also included notations for pauses, verbal and non-verbal units and incidents in the transcripts. The FOLKER transcripts were segmented into speaker turns.

### Normalisation

We used the tool OrthoNormal^18^ to add a normalisation layer for transcribed tokens. In the normalisation process, we compared the transcriptions to the standard variety that participants indicated as the language they spoke in the questionnaire, since BCMS has four different standard varieties. For instance, if a participant indicated “Croatian” as his/her language and used the eastern variant *tačno* instead of *točno* (‘correct’, ‘exactly’), as the word is said in the Croatian standard, we normalised it as *točno*, although *tačno* is correct in other BCMS standard varieties. This allows assessing how close their language use is to the standard varieties they name or regard as their own. We also normalised hesitation (*ähm, äh, ääh*, etc.) and acknowledgement tokens (*hmhm, mhm, mh*, etc.) to *äh* and *hm* respectively.

We also used OrthoNormal to annotate truncations, non-BCMS words, invented words, stutter, unclear words, and elongations (see Table 1). In doing so, we used conventions presented in Winterscheid et al. (2019). In total, 2538 tokens were affected by the normalisation (12.7%).

**Table 1:** Normalising conventions in XML transcripts (FLN-transcripts)

Symbol	Description	Example
%	Truncations	pre (prema %)
§	Non-BCMS Non-existing words	Blumenkohl (Blumenkohl §) klem (§)
#	Stutter	p p prema (p # p # prema)
+	Unclear syllables	+++
$	Elongation	premaa (prema $)
n=“{}”	Normalisation to the standard variety	<w n=“lijepo”>lipo</w>

### TEI encoding

We converted XML transcripts produced by OrthoNormal into TEI-XML encoded transcripts, following the TEI guidelines for transcriptions of speech implemented in the Corpus of Serbian Forms of Address (Lemmenmeier-Batinić, 2021, pp. 131–132).^19^ In addition, we added the element <seg>, in which we stored non-BCMS words (@type=“non-bcms”), invented words (@type=“non-word”), elongations (@type=“elongation”), hesitation tokens (@type=“hesitation”) and acknowledgment tokens (@type=“acknowledgment”).

We tagged the corpus with the Serbian model of the CLASSLA-StanfordNLP tagger (Ljubešić & Dobrovoljc, 2019)^20^ and stored the annotations for lemmas (@lemma), universal part-of-speech tags (@pos),^21^ MULTEXT-East Serbo-Croatian morphosyntactic specifications^22^ (@ana), and normalisations (@norm) in the <w> element (see Example 1).^23^


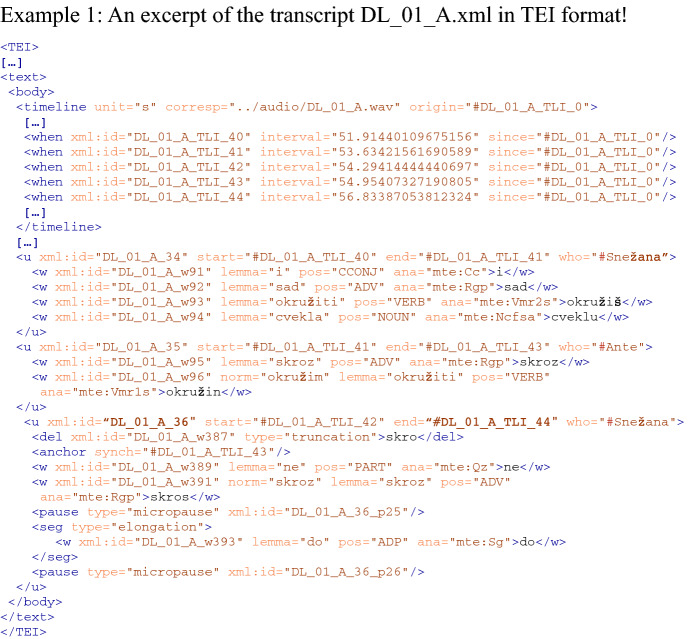


## Web interface

In this section, we present the web interface of the corpus, which allows for a preliminary assessment of lexical, phonetic, and morphosyntactic features of heritage BCMS. Upon user registration, it allows users to view different transcript versions and already implemented annotations, make a pre-selection of transcripts through metadata filters, and create, store and share custom annotations directly on the corpus platform. To enable these functionalities, we elaborated the metadata so that it can be searched and filtered, converted the TEI transcripts into html files, and enriched them with annotation-related functionalities.

### Metadata view

**Fig. 3:**
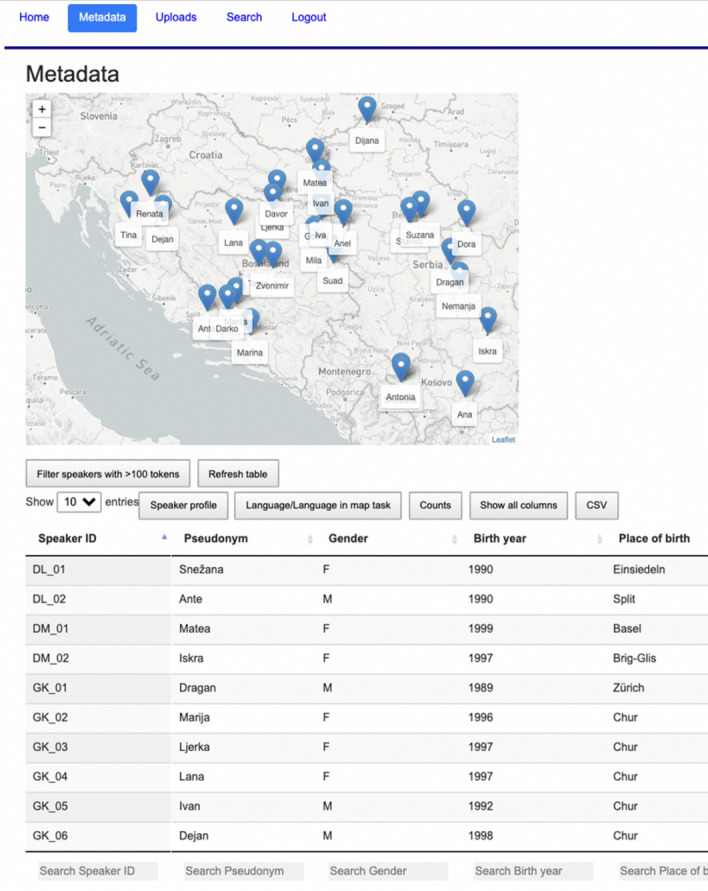
Interactive table and map

**Fig. 4:**
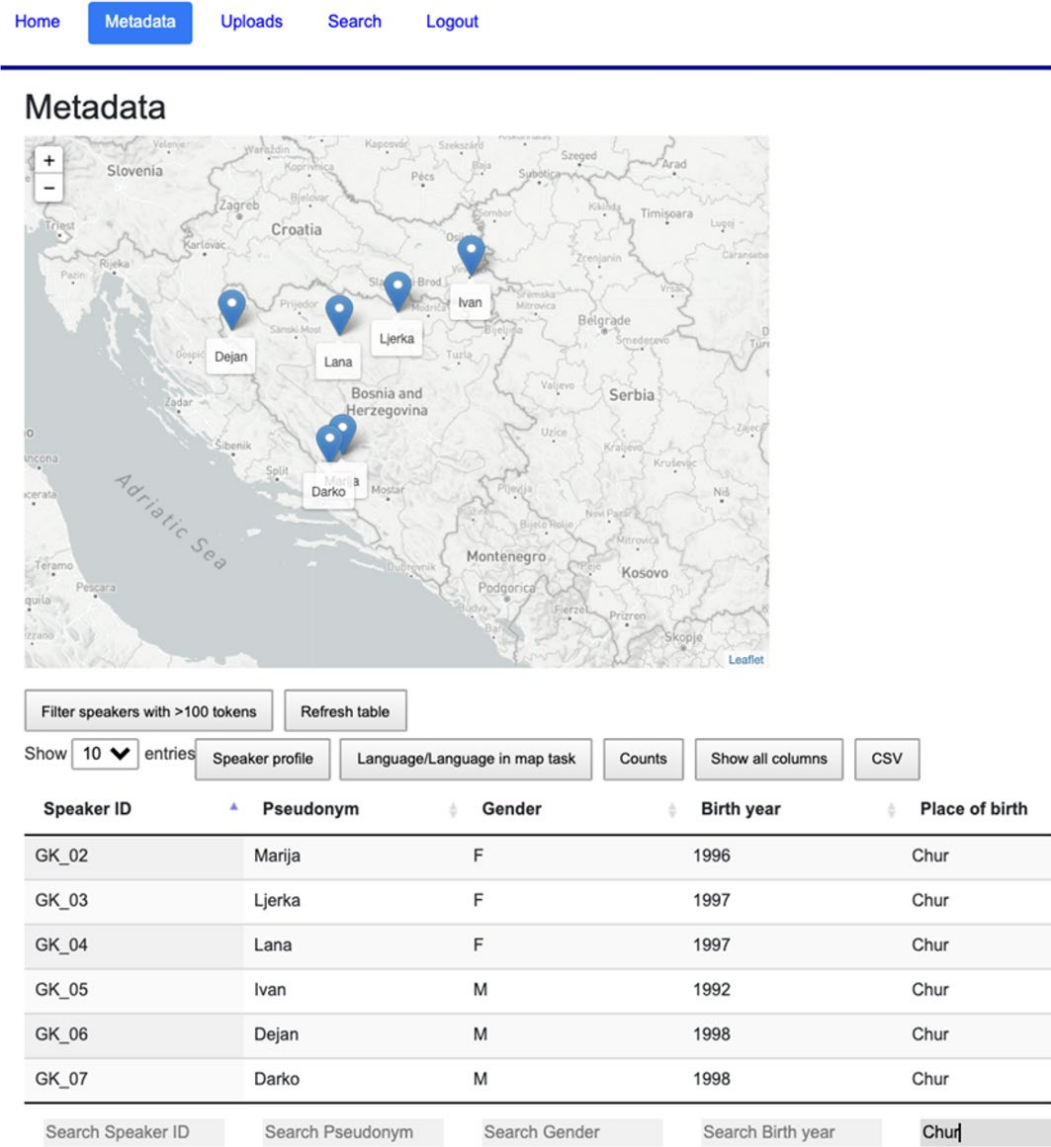
Filtering the table and map on participants born in Chur

Speaker metadata collected in the questionnaire as well as the corpus counts for each speaker are presented in an interactive table created with the jQuery plug-in DataTables^24^ (see Fig. 3). The table is populated with the metadata stored in JSON format. Each column can be sorted and filtered using regular expressions. The table filter is synchronised with the map view representing the place of origin for each speaker, which gets updated according to the current table selection (see Fig. 4).^25^

**Fig. 5:**
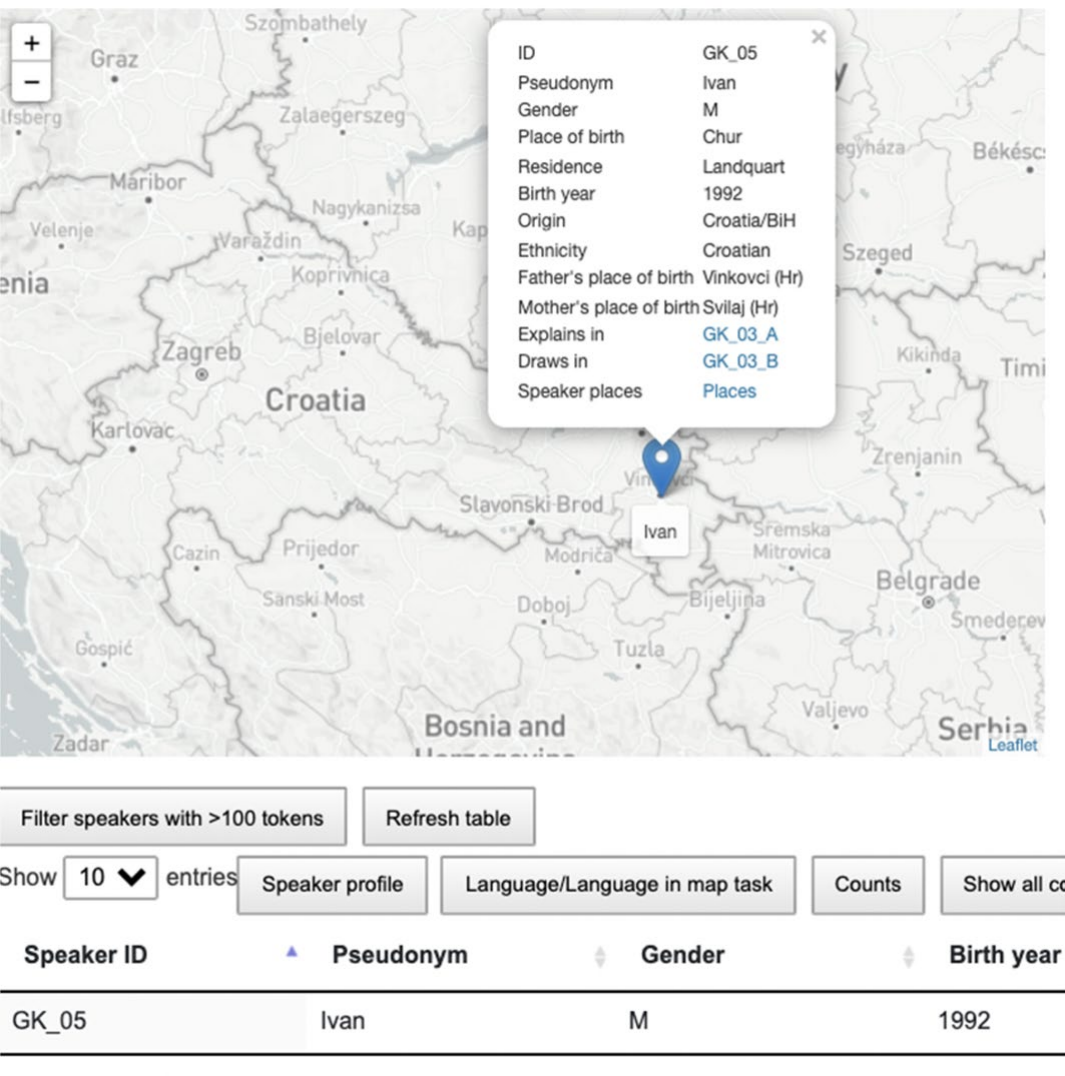
Pop-up window for the participant with the pseudonym *Ivan*

**Fig. 6:**
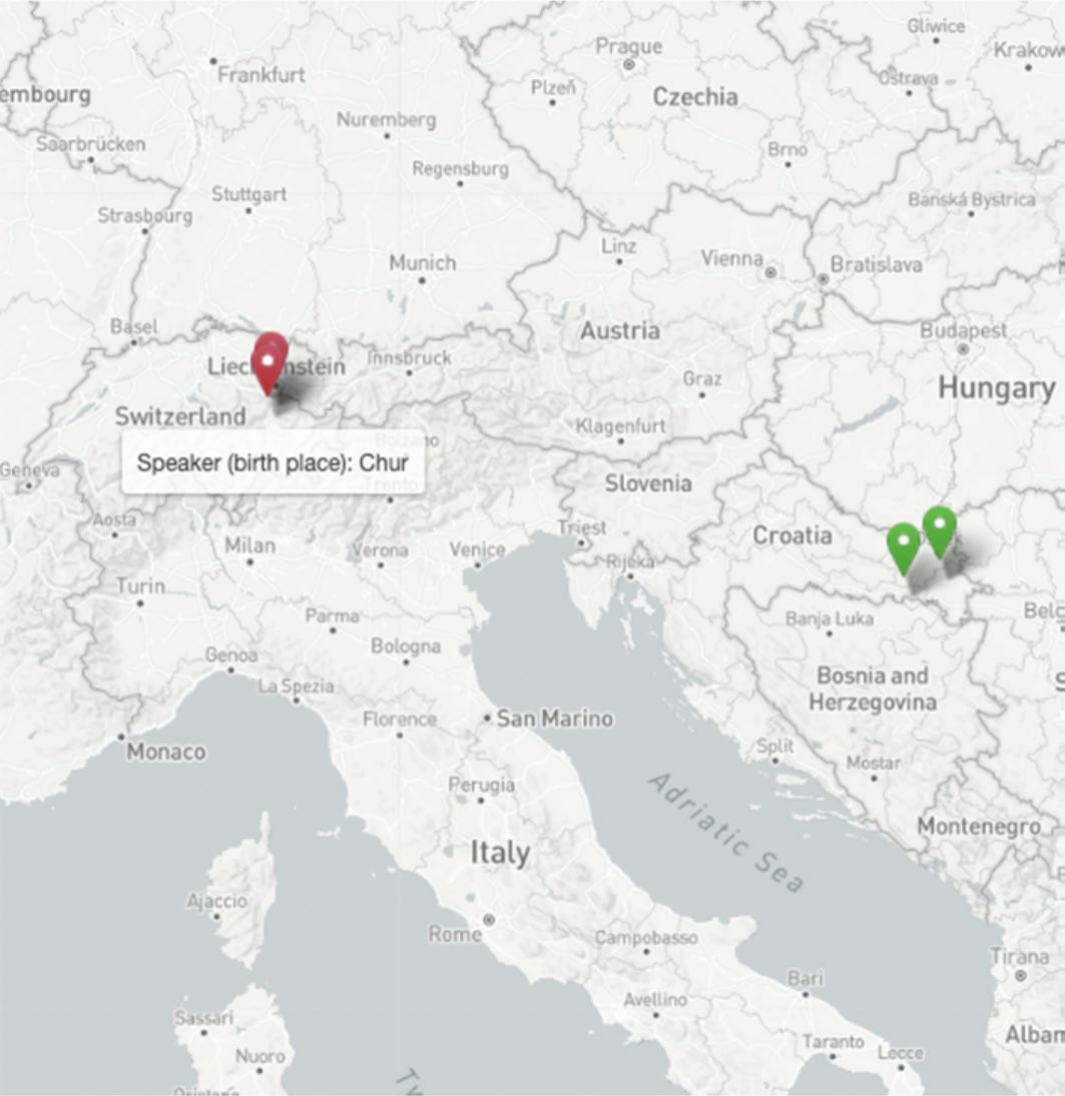
Birth place and residence of the participant (red), and birth place of both parents (green). (Color figure online)

As shown in Fig. 5, when the speaker’s location (marker) is clicked, the metadata table gets filtered on that speaker, and a pop-up window appears containing basic speaker information, as well as links to the transcript view and view of all the relevant places for that speaker (place of birth and residence of the speaker, and place of birth of both parents, see Fig. 6).

We divided the metadata table content into three parts to allow for easier search: *Speaker profile, Language/Language in map task*, and *Counts*. Metadata in *Speaker profile* and in *Language/Language in map task* was taken from the questionnaire, while corpus counts in *Counts* were added after the normalisation step. Prior to the implementation, information about the speaker profile (birth place, birth year, parents’ origin, education, media usage, etc.) and about their language use (dialect, accommodation, language attitudes, reflection on language use in the map task, etc.) has been made consistent^26^ and translated into English in order to fit with the main language of the web interface. In answers that refer to a 5-point Likert scale, “1” stands for “disagree/false”, and 5 stands for “agree/correct”.

**Fig. 7:**
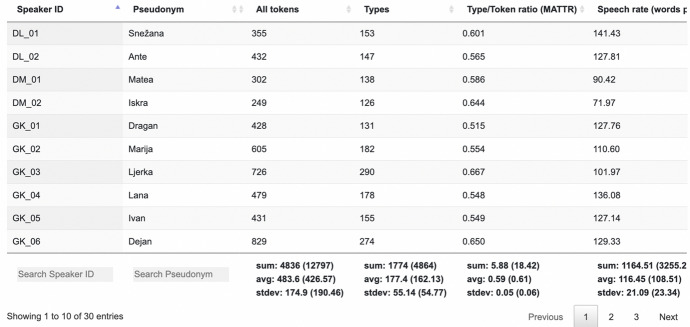
An excerpt of the *Counts* section in the metadata table

The *Counts*-section comprises token and type count, type/token ratio,^27^ speech rate, number of map task images that participants knew in BCMS, and absolute and relative counts of non-BCMS tokens in the transcripts, normalised tokens, pauses, hesitations and elongations.^28^ For each corpus count we also implement the calculation of sum, average, and standard deviation of currently selected data (see Fig. 7), as well as all data in the set (in parentheses). The metadata table can be exported as a text file in CSV format.

### Transcript view

**Fig. 8:**
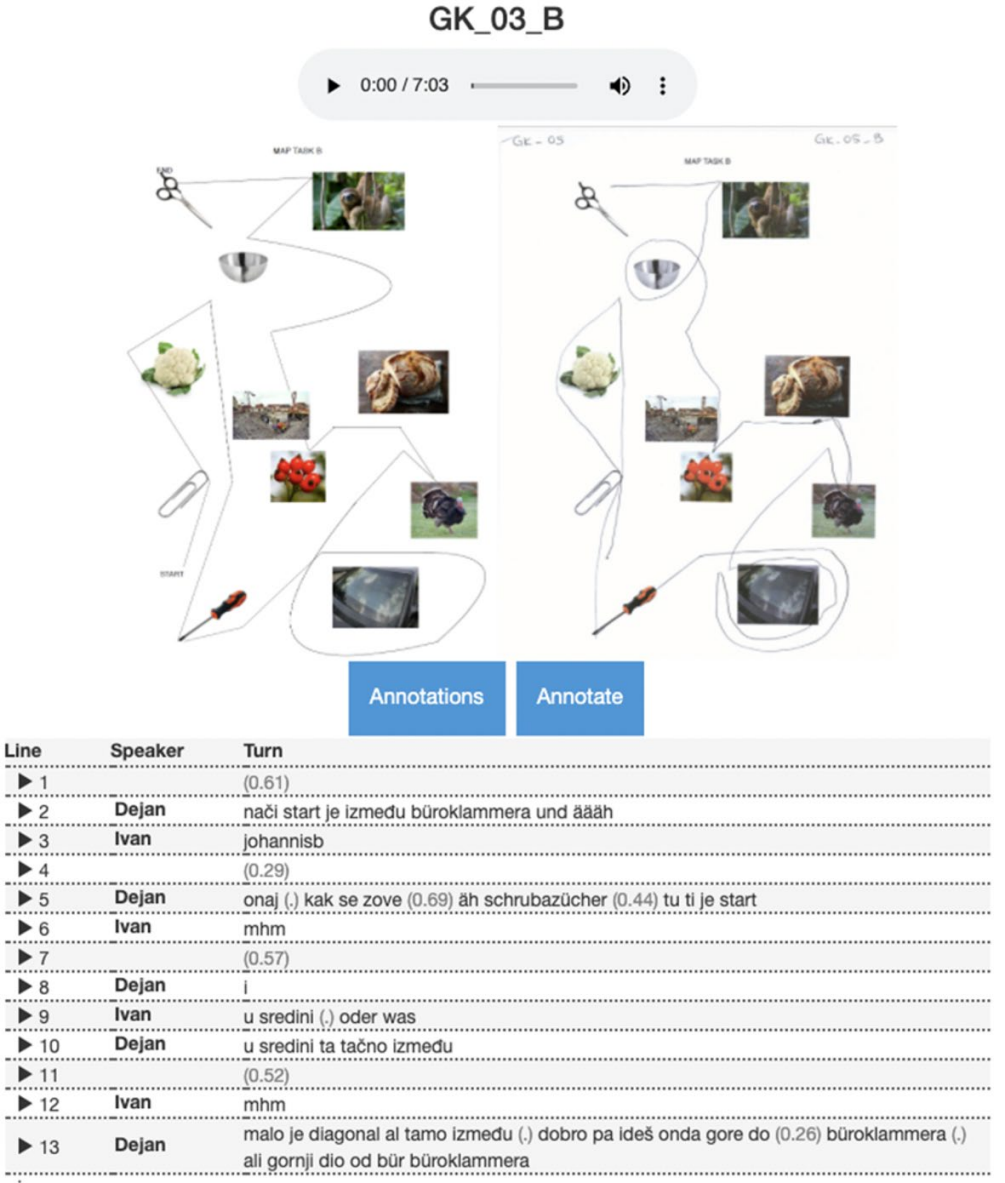
Transcript view

**Fig. 9:**
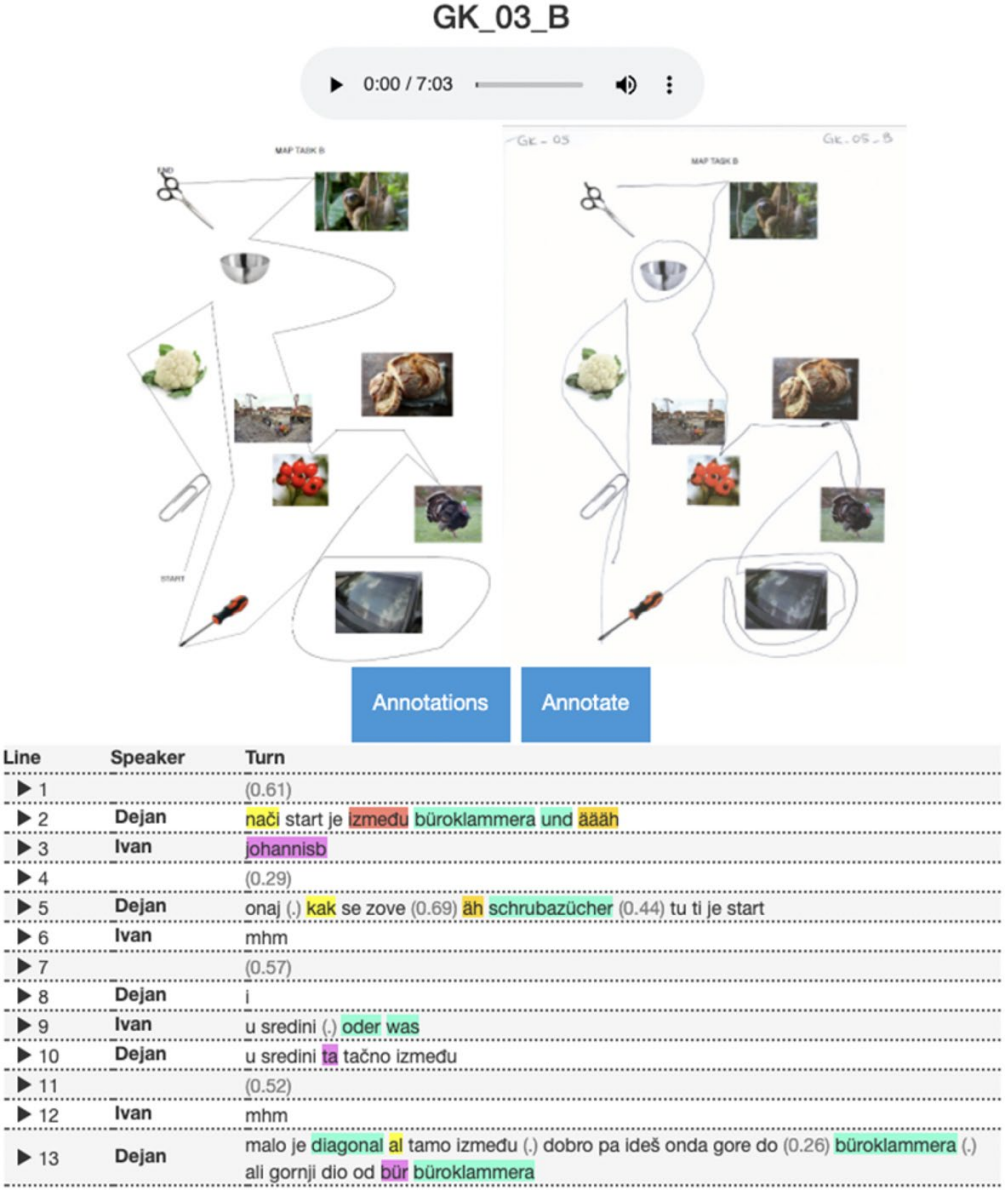
Transcript annotations

The transcript view consists of the pictures of the original map task, the solved map task and the transcript of the conversation (see Fig. 8). Each turn is aligned with the audio segment and can be displayed on mouse click on the respective turn number. On click on the participant’s pseudonym the user gets redirected to the metadata view as shown in Fig. 5. To facilitate the reading of the transcript, we added a background colour to the segments that have been encoded in the normalisation step. Pauses are marked in parentheses: pauses shorter than 0.2 s are marked as (.), while longer pauses are reported in seconds, just like in the FOLKER output. Annotations created in the normalisation process can be viewed either separately or all together on click on *Annotations* button (see Fig. 9). They can be exported as CSV files.

The transcript view has been created with an XSL-stylesheet that can be retrieved at the corpus homepage (section *Transcripts*), and further edited for transforming own TEI-encoded transcripts of speech. We used the same minimally adapted XSL-stylesheet to create a HTML version of the Corpus of Serbian Forms of Address.^29^ All transcripts are additionally made available for download in the start-page in following formats: TEI, FLK (raw FOLKER transcripts), FLN (normalised transcripts), TEI (tagged transcripts) and TextGrid (for the use with Praat^30^).

**Fig. 10:**
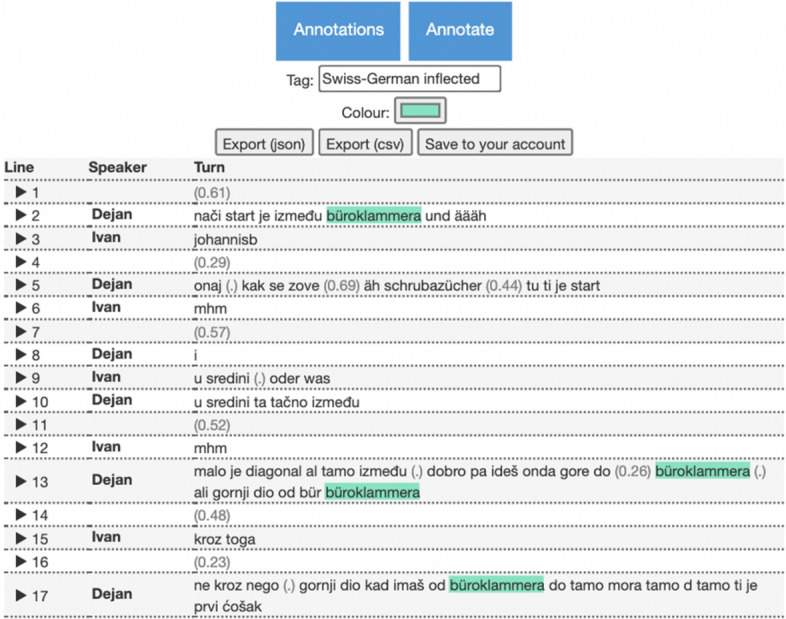
Custom annotations for the transcript GK_03_B

By clicking on the button *Annotate* users can add their own custom annotations with the custom tag and colour by selecting one or more tokens (see Fig. 10).^31^ Multi-word annotations are also supported. The annotations can be either exported as CSV and JSON files or saved directly in the user profile. Exported annotations in JSON format can be uploaded and edited in the Uploads page (see Sect. 4.3).

### Custom annotations view (uploads)

**Fig. 11:**
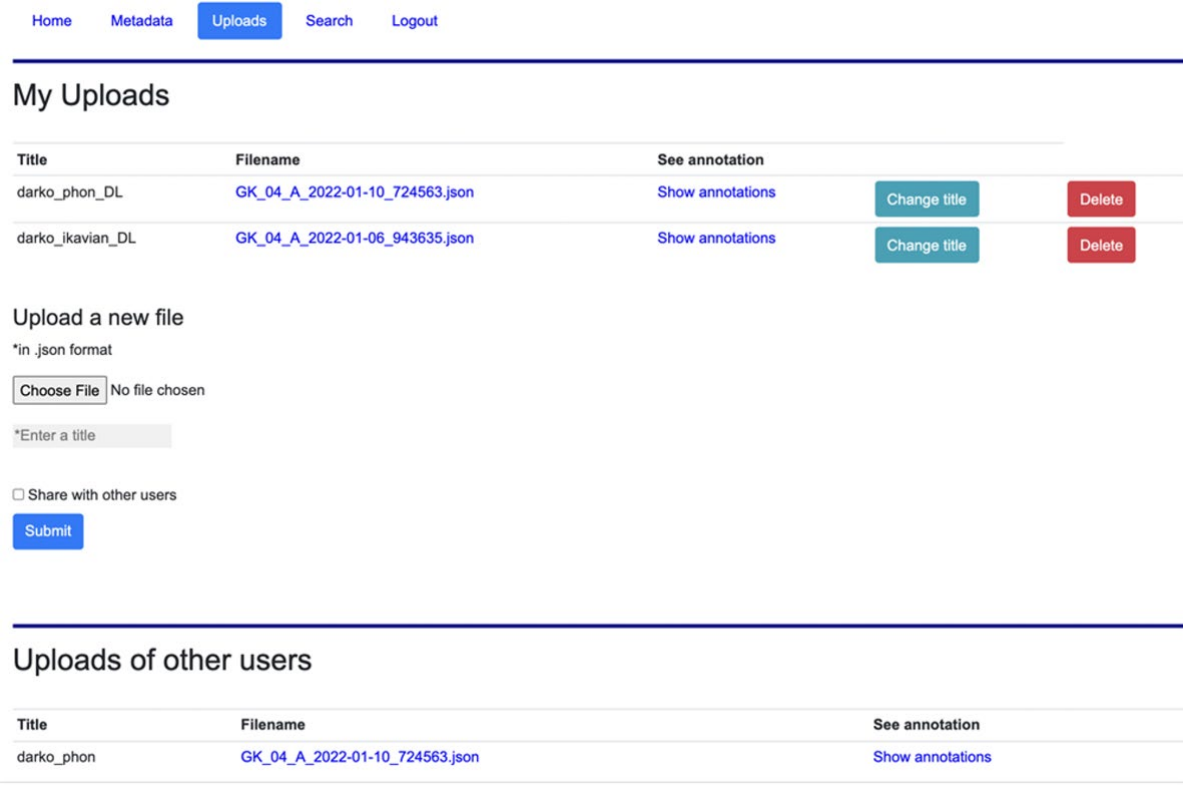
Uploads page

Custom annotations are stored in the mongoDB^32^ database and accessed via Python web framework Flask.^33^ In the Uploads page, users can view their custom annotations and share them with other users. They can also upload and further edit their own annotations as well as shared annotations of other users by clicking on *Show annotations*, which redirects the users to the transcript view with custom annotations (see Fig. 11).

### Search view

**Fig. 12:**
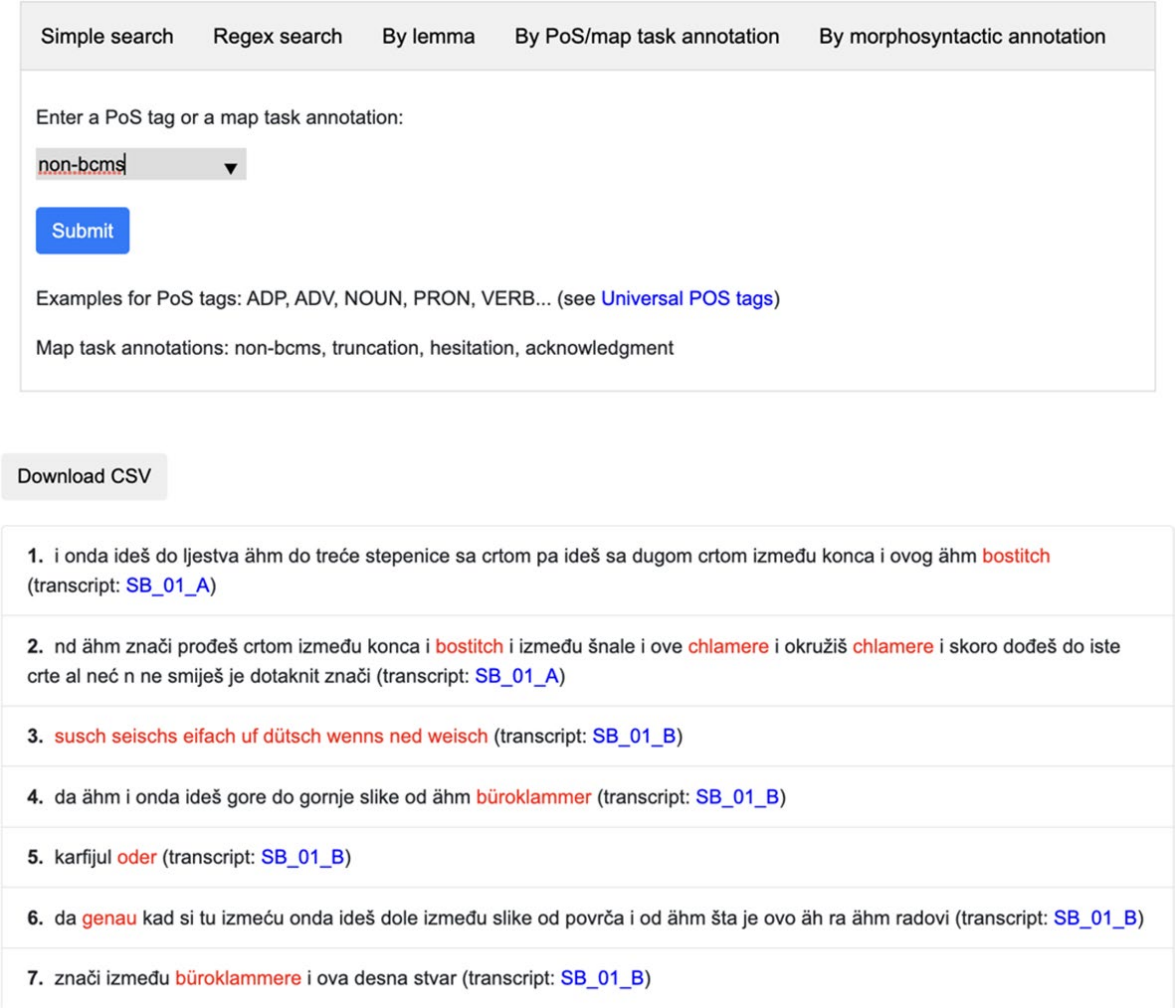
Search view: an excerpt of the hits for the search of all non-BCMS tokens

The search view allows for querying the word form (tokens), lemmas, universal parts of speech, morphosyntactic annotations, and by map task annotations added in the normalisation step (non-BCMS tokens, truncations, hesitations and acknowledgment tokens). We implemented simple as well as regular expression search for word forms. After user submits the query, the results are retrieved from the database and presented in the context of the whole turn (see Fig. 12). For each result, a link to the respective transcript is provided. The search results are available without registration, but users have to be registered to access the transcripts. Results can be exported in CSV format.

### User flow diagram

**Fig. 13:**
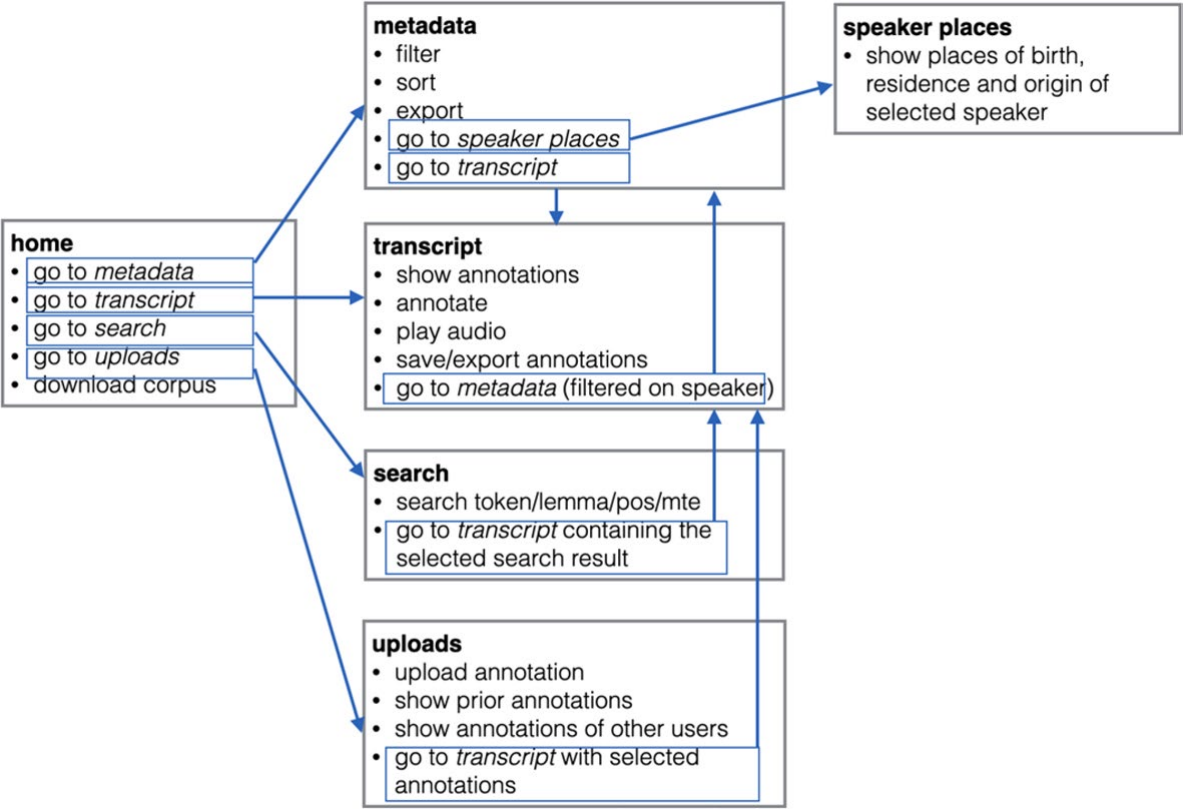
User flow diagram

The functionalities presented in prior sections are summarised in the user flow diagram in Fig. 13. The diagram shows the structure of the web-interface, the links between different web pages, as well as main functionalities provided on the corpus interface.

### Corpus access

Upon user registration, the corpus is accessible at https://maptask.slav.uzh.ch. For long-term deposit, we plan to store the corpus on CLARIN.SI. Current work, as well as demo code for transcript annotations is documented at the GitLab Repository of ZuCoSlaV corpora (Zurich Corpora of Slavic Varieties).^34^ The corpus is licensed under a Creative Commons Attribution-NonCommercial-ShareAlike (CC BY-NC-SA).^35^

## Case study: heritage BCMS used by a pair of siblings

Although they are brought up in the same household, siblings can have great differences in heritage language proficiency (Aalberse & Muysken, 2013, p. 6). While Baker (2007, p. 56) argues that a younger child is simply integrated in the decisions about language that are already established in the family, Kheirkhah & Cekaite (2018) challenge that by claiming that siblings may have different experiences of the heritage language related to factors such as the age of migration, social environment and their aspirations. It is often argued that the eldest child speaks the heritage language most native like (Aalberse & Muysken, 2013; Jarovinskij, 1995; Shin, 2002; Wong Fillmore, 1991) since they receive more speech input from the parents. The school entry of the eldest child usually implies the introduction of the dominant language in the family. Commonly, younger siblings receive less input in their heritage language, which also impacts their active use of the heritage language. Siblings tend to interact among each other using the dominant language, i.e., the language spoken at school (Döpke, 1992). This has also been observed by Romić (2016, p. 200) regarding the second-generation heritage BCMS speakers in Germany, where only 6% of the survey participants (n = 103) report to always speak their heritage language with their siblings. Romić (2016) also observed that second-generation speakers speak BCMS commonly with other heritage speakers: 13% of them reported to speak BCMS with their friends from former Yugoslavia (which is more than they reported to speak BCMS with their siblings). Mayer & Lemmenmeier-Batinić (in preparation) observed that 28% of second-generation speakers living in Germany, Austria, and Switzerland report to always speak BCMS with their siblings, and 30% to always speak BCMS with people from former Yugoslavia (n = 175).^36^

In the following sections, we use the BCMS Map Task corpus platform to conduct an exemplary case study of language use by a pair of siblings. After presenting the participants and corpus measurements, we analyse their use of lexical, morphosyntactic, and phonetic features.

Within the lexical domain, we study the use of *lexical transfers* from (Swiss-)German into BCMS (see Ščukanec, 2021, p. 263). An example for a lexical transfer from German to BCMS is *anrufati* (from *anrufen* ‘to ring sb.’, see Ščukanec et al., 2021, p. 118). Since map tasks are picture naming tasks, we expect to find frequent use of lexical transfers from Swiss–German. The adaptation of lexical transfers to BCMS morphosyntactic and phonetic systems is expected to be variable in second-generation speakers (see Ščukanec et al., 2021, pp. 125–126) and to be common among “more proficient heritage speakers” (see Brehmer, 2021, p. 33). In addition, we measure the lexical proficiency by counting the number of correctly named images in the map tasks, and comparing it to other participants’ results.

Regarding morphologic and syntactic features, we focus on verbal aspect and word order patterns, which are reported to be vulnerable domains for heritage speakers (see Brehmer, 2021, pp. 30–32; Polinsky & Kagan, 2007). Several studies report the loss or confusion of verbal aspects in Slavic heritage languages (see Hill, 2014, p. 2128), as well as on influence of dominant word order patterns of the majority languages on the heritage language (Hansen et al., 2013; Laskowski, 2014).

Regarding the domain of phonetics, we focus on the realisation of the alveolo-palatal affricate /dʑ/ (đ).^37^ Given that this consonant is missing in Swiss–German, we expect deviations in its realisation (see Brehmer, 2021, p. 25). Since the devoicing of affricates has already been observed in Italian spoken by second-generation heritage speakers living in German-speaking Switzerland (see De Rosa & Schmid, 2000), we hypothesise that the speakers might have difficulties in distinguishing the alveolo-palatal /dʑ/ from its voiceless counterpart /tɕ/, which is also present in BCMS.

### Participants

**Fig. 14:**
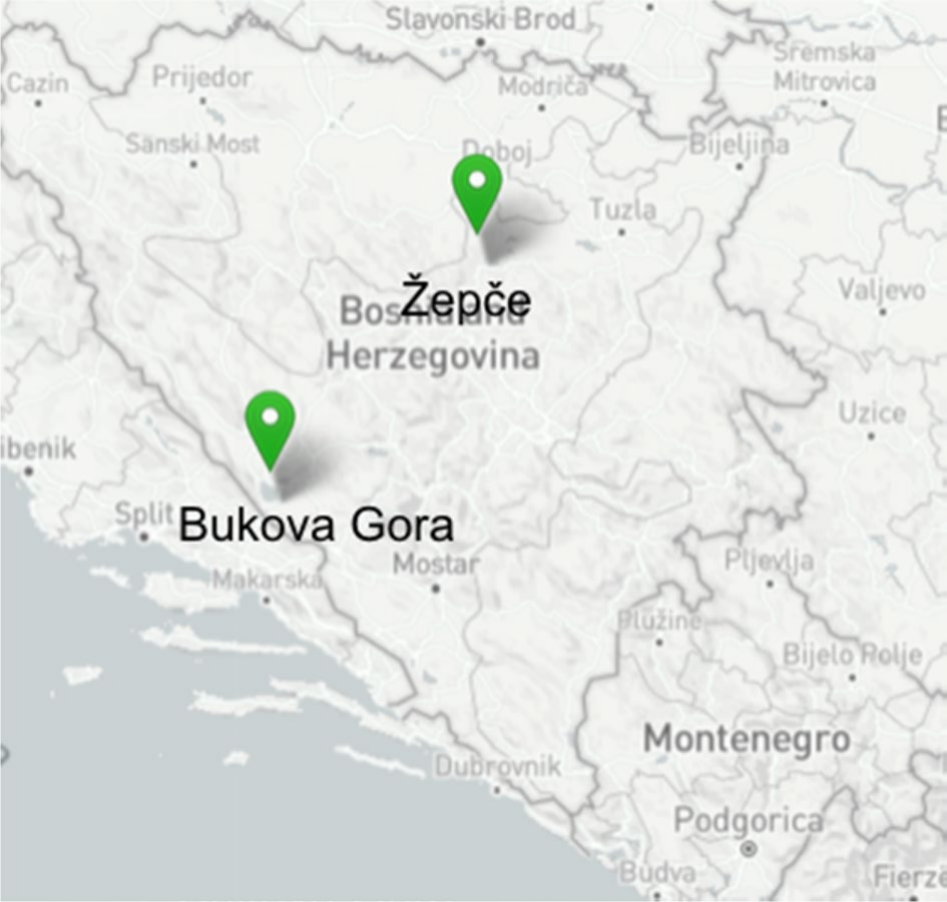
Places of parents' origin of the two siblings

The speakers Marija* and Darko* were born in Chur and lived in Landquart at the time of recording (both towns are situated in the southeast of Switzerland). At the time of recording, Marija was 24, and Darko 22 years old. Maria is the eldest child in the family, Darko the second child, and they also have a younger brother (16 years old at the time of recording). Their parents are Croats from Bosnia and Herzegovina. Their father is born in Bukova Gora, where he spoke Ikavian, and their mother in Žepče, where she spoke Ijekavian (see Fig. 14). Marija attended university, and Darko has a high school diploma.

### Map task setting

Marija explained map task B to a male participant, Dragan*, whose parents originate from Svilajnac (Serbia), and whom she did know before the task. Darko explained map task A to a female participant Marina*, whose parents are Croats from Grabovčići (BiH) and Pokrajčići (BiH), and whom he already knew before. Hence, the setting is not identical, and the participants’ performance might vary depending on the *interlocutor effect* (see Son, 2016).^38^ Nevertheless, since the two map tasks are comparable in content and difficulty level, we assess that the speech material provided in the conversations is adequate for a further analysis of their language use.

### Language profiles

As shown in Table 2,^39^ both siblings name the variant they spoke in the map task as Croatian and report to have spoken the Štokavian dialect with the Ikavian *jat* vowel reflex, the same variant they report to speak at home. As expected, they both report to frequently speak BCMS with their parents (4 on a scale from 1 to 5). Surprisingly Darko, the younger sibling, reports to speak BCMS with his siblings more often than Marija reports doing so (Darko: 4; Marija: 3). He also reports to speak more often BCMS with “people from former Yugoslavia” than Marija does (Darko: 5; Marija: 3). Darko specifies never to speak BCMS with his partner, while Marija leaves this question unanswered. As expected, both siblings report to mix their heritage language often with Swiss–German (Marija: 4; Darko: 5). It goes without saying that the scale is subject to personal interpretation (the participants might mean the same, but assess their answer as 4 or 5).

**Table 2:** Language profiles of Marija and Darko

	Question	Marija (age: 24)	Darko (age: 22)
Reflections about language	First language	Croatian	Croatian
	What language did you speak in the Map Task?	Croatian	Croatian
	How did you speak (dialect)	Štokavian	Štokavian
	How do you speak at home (dialect)	Štokavian	Štokavian
	How did you speak (pronunciation)	Ikavian	Ikavian
	How do you speak at home (pronunciation)	Ikavian	Ikavian
	I always speak BCMS with my partner	N.A.	1
	I always speak BCMS with my parents	4	4
	I always speak BCMS with my siblings	3	4
	I always speak BCMS with people from former Yugoslavia	3	5
	I often mix the language of the country I live in and my heritage language	4	5
Experience and socialisation in BCMS	Attended classes in BCMS	No	No
	Media consumption in BCMS	5	2
	I often go to my country of heritage	4	5
	I have regular contact with people in my country of heritage	4	3
Language attitudes	I think it's important not to mix the languages (e.g. Swiss–German–BCMS)	3	1
	It is important to me to pass the heritage language on to the next generation	5	5

**Table 3:** Quantitative information on the language used in map tasks

Counts	Marija (age: 24)	Darko (age: 22)	Average participant
Tokens (when giving instructions)	489	442	352.07
Tokens (when taking instructions)	116	159	74.50
All tokens	605	601	426.57
Types	182	185	162.13
Type/token ratio (MATTR)	0.554	0.568	0.61
Number of normalised tokens (rel)	11.1 (abs: 64)	13.0 (abs: 72)	6.60
Nr. of non-BCMS tokens (rel)	4.5 (abs: 27)	8.0 (abs: 48)	3.53
Speech rate (words/min)	110.60	117.61	108.51
Number of pauses (rel)	19.2 (abs: 116)	20.3 (abs: 122)	23.52
Number of hesitations (rel)	2.1 (abs: 13)	0.8 (abs: 5)	3.87
Number of elongations (rel)	0.3 (abs: 2)	0.0 (abs: 0)	2.04

The relative values (‘rel’) stand for the calculated number of occurrences of a particular phenomenon within a 100-words-window

Regarding the experience in BCMS, both siblings report that they did not have classes in BCMS, but Marija self-evaluates her media consumption in BCMS as much higher than her brother does.^40^ While Darko reports to go to their country of heritage more frequently than Marija does, according to her estimation, Marija has more regular contact with people in Bosnia and Herzegovina.

Both siblings absolutely agree with the statement that it's important for them to pass the heritage language on to the next generation. While Darko strongly disagrees with the statement that it’s important not to mix languages (e.g. Swiss–German–BCMS), Marija has less strong opinion on this matter and neither agrees nor disagrees.

In summary, the two participants show to have a meta-knowledge regarding the dialectal variant they speak, which is not always the case for heritage speakers (31% of the heritage speakers in the survey by Mayer & Lemmenmeier-Batinić [in preparation] report not to know which dialect they speak). According to their reports, they used the same variants they speak at home also in map task communication. Their use of BCMS in communication with their parents, siblings and other people is relatively in line with theoretical and empirical considerations on heritage speakers mentioned in Sect. 5. The analysis of the metadata showed that both siblings have close ties with their heritage country, that they use BCMS in their private and social life, and that they also aim to pass it to the next generation. In such, they are typical representatives of second-generation BCMS heritage speakers living in a German-speaking country (see Mayer & Lemmenmeier-Batinić, 2021).

### Corpus measurements

In Table 3 the quantitative information regarding the language use in the map tasks is given. It shows that the two siblings have relatively similar language profiles. They even uttered nearly the same number of tokens in the map task conversations (605; 601), which is more than average (426.57). However, the quantitative information calculated from the normalised transcripts suggests that in comparison to an average participant the siblings used more non-standard words and more non-BCMS words. They both have a slightly faster speech rate than the average, and a lower number of pauses, hesitations and elongations. Regarding Darko, the latter is possibly due to the fact that he used more than twice as much Swiss–German words than an average participant, and hence spent less time hesitating and trying to find the correct BCMS expression. His self-evaluation regarding his frequent mixing of heritage and dominant languages is reflected in his frequent use of Swiss–German words that are directly inserted into BCMS turns, without being morphologically or phonetically adapted to it (see Sect. 5.5.1).

### Analysis of language use

According to the observations shown in Sects. 5.1–5.4, we expect Marija to exhibit a higher level of proficiency in BCMS than Darko because she is the eldest child in the family, and because her self-evaluation on consuming BCMS media is much higher than her brother’s (see Sect. 5.3). However, the difference in heritage language proficiency between the two siblings is not expected to be large: on one hand, Darko reports to always speak BCMS with people from former Yugoslavia, and exhibits an overall similar language profile as Marija: in most questions they present none or only 1-point-difference on a Likert scale from 1 to 5 (see Table 2). On the other hand, their age difference is relatively small (2 years), so the difference in length of exposure to the heritage language is also small. Corpus measurements regarding the conversations by the two siblings draw a similar picture, with the most prominent difference being the higher relative number of non-BCMS tokens in Darko’s turns, which supports the assumption that Darko has a lower lexical proficiency in BCMS than Marija.

#### Lexical observations

The siblings showed difficulties in naming map task images in BCMS. They both named 4 out of 10 images correctly, which is lower than an average participant (the average number of correctly named images is 5.53).^41^ However, while both siblings often referred to the images without naming the objects themselves (by just saying *slika* ‘image’), they used different strategies when confronted with difficulties in lexical retrieval. Darko mostly inserted Swiss–German lexical transfers directly in BCMS sentences without adapting them to BCMS grammatical system. For instance, the insertion *schloss* (‘lock’), which is used in instrumental, would have been inflected as *schlossom* (‘lock’-INS) in BCMS, see Example 2.^42^

Example 2: Excerpt from the transcript GK_04_A (Swiss–German words are represented in italics)^43^

[...] na *höche* malo ispod on-e slik-e sa *schloss*

    on height a little below that-GEN image-GEN with lock

‘[...] on the height a little bit below that image with the lock’

The use of morphologically non-integrated lexical transfers is consistent in Darko’s turns: out of 14 lexical transfers that require case marking in BCMS, the case is marked only in one of these, as Darko inflects the word *schloss* in genitive to *schloss-a* (‘lock’-GEN), as a masculine singular noun would be inflected in BCMS. Overall, Darko uttered 31 lexical transfers, which is more than any other participant. In contrast to her brother, Marija never used lexical transfers from Swiss–German, but she tried to explain the map task path with her own paraphrases in BCMS, as shown in Example 3, in which she paraphrases the word ‘paperclip’. In other cases, she used hypernyms for the words represented in the images (like *bird* for *turkey* or *animal* for *sloth*).

Example 3: Excerpt from the transcript GK_01_B (paraphrase in italics)

[...] i onda ideš .hh (0.45) prema doli (.) u sredinu između *onog što drži listove* i između šipka

‘[...] and then you go .hh (0.45) down (.) between *the thing that holds the sheets together* and the rose hip’

Darko also used hypernyms, but even in those cases he sometimes used Swiss–German words like *frucht* (‘fruit’) for *beetroot*. Marija used Swiss–German only in meta-comments about the map task, in which she expressed her difficulties in retrieving the right word, as shown in Example 4.

Example 4: Excerpt from the transcript GK_01_B (‘nothing comes to my mind’)

[...] i onda o tu ((smije se, 0.83s)) ideš (0.6) ehm (.) pored ehm ((nejasno)) (0.31) ehm (.) .hh (1.91) ono .hh ((smije se, 2.2s)) (0.62) ((smije se, 1.45s)) .hhh *sorry aber miar fallt gar nüt i* .h (0.29) ehm .h

‘[...] and then from there ((laughs, 0.83s)) you go (0.6) ehm (.) near ehm ((unclear)) (0.31) ehm (.) .hh (1.91) that .hh ((laughs, 2.2s)) (0.62) ((laughs, 1,45s)) .hhh *sorry but nothing comes to my mind* .h (0.29) ehm .h’

Non integrating other-language-items to a heritage language as Darko is the opposite of what is expected from a proficient heritage speaker (see Brehmer, 2021, p. 33). However, Marija’s use of avoidance strategies reveals difficulties in lexical retrieval as well.

#### Morphosyntactic observations

One recurring morphosyntactic deviation in Darko’s utterances is the consistent use of the conjugation *dok* (‘until’) with the imperfective present form of the verb *biti* ‘to be’ (*dok si*-PRS.2SG) instead of the negated perfective form *budeš* (*dok ne*-NEG budeš-PRS.2SG), which was encountered five times in the transcript (see Example 5).^44^

Example 5: Excerpt from the transcript GK_04_A (*dok si*)

[…] dalje vuči dole *dok si* ispod te (1.23) ispod te äh (0.29) kutlače (.)

‘[…] draw further down *until you are* below that (1.23) below that äh (0.29) soup spoon (.)’

This use of verbal aspect and the missing negation is possibly due to the process of *reduction*, i.e., simplification of syntactic structures in heritage languages, comparable for instance to the loss of double negation in Swedish Polish (Laskowski, 2014, p. 119). Not to neglect is also a possible (Swiss-)German influence, since in (Swiss-)German there is no negation after the conjugation *bis* (‘until’) used in this sense.

Other participants also showed deviations in verbal aspect after subordinate sentences introduced with the conjunction *dok*: the search of all turns with *dok* (*Search* page) shows that out of a total of 20 occurrences, *dok* never occurred followed by a negated form, but always followed just by a perfective or imperfective form of the verb *biti* (*budeš/si*). The non-standard use of imperfective form in utterances such as (5) is not surprising given the loss or confusion of verbal aspects in Slavic heritage languages that was reported in several studies (see Hill, 2014, p. 2128).

In contrast, the placement of the verb in clause-final position (see Example 6) is not ungrammatical, but rather “perceived as somewhat odd” in standard BCMS (Hansen, 2018, p. 5). This phenomenon occurred only twice in Darko’s transcript, so it is hard to talk about a (deviant) pattern.

Example 6: Excerpt from the transcript GK_04_A (verb position)

[...] ali na desn-u stran-u trea-š crt-u *vuč*

    but on right-ACC side-ACC you need-PRS.2SG line-ACC *to draw-INF*

‘[...] but you have to draw the line to the right’

We detected no recurring patterns regarding deviations in verbal aspect and word order in Marija’s map task conversation. However, it has to be noted that Marija never used the construction with *dok* (‘until’), so we cannot know how she would use the verbal aspect in utterances like (5).

Regarding the detection of morphosyntactic patterns in the corpus, pre-annotated features such as *non-standard words* can be helpful in case when a particular word is non-standard because of a non-standard morphology (for instance, *do donj-og-*GEN.SG.M., instead of *do donj*-*eg*-GEN.SG.M., ‘until the lower’). Syntactic deviations such as word order are however not pre-annotated and have to be searched either by examining the transcript in the *transcript* view or by querying the transcripts on relevant phenomena in the *search* page.

#### Phonetic observations

As expected, both speakers showed deviations in the realisation of /dʑ/. However, the siblings showed no difficulties in distinguishing /dʑ/ from its voiceless counterpart /tɕ/, as it was hypothesised. Instead, they often uttered a less palatal sound, which can be either described as post-alveolar* /*dʒ/ (dž), or falling into the spectrum between /dʑ/ and /dʒ/*.*^45^ Different realisations repeatedly occurred within the same word *između* ‘between’, as in Examples 7 and 8, where *između* is first realised as [izmedʒu] and than as [izmedʑu].

Example 7: Excerpt from the transcript GK_01_B (*dʒ*)

[…] i onda .h (.) ideš prema desno gori (.) do sredinu *između* [izmedʒu] (.) kruha i one ptice

‘[…] and then you go up to the right to the middle *between* the bread and that bird’

Example 8: Excerpt from the transcript GK_01_B (*dʑ*)

[…] i onda (0.21) do s u sredinu tamo *između* [izmedʑu] kruha i te ptice

‘and then to the middle *between* the bread and that bird’

Interestingly, the tendency to realise post-alveolar /dʒ/ instead of alveolo-palatal* /*dʑ/ cannot be explained as a *direct* influence from Swiss–German, since both sounds are absent in Swiss–German phoneme inventory. While in homeland BCMS the difference between the affricates /dʒ/ and /dʑ/*,* is disappearing in some regions as well, the tendency there is rather to replace /dʒ/ by /dʑ/ (see Peco, 2000; Halilović et al., 2009, p. 117).^46^ This development is hence worth further investigation, also because different deviations in realisation of /dʑ/ have been evidenced by other speakers as well.^47^

For finding and evaluating the use of the alveolo-palatal* /*dʑ/, we used the *Search* page (with which we could find the segments by using *Regex search*) and the *transcript* view for evaluating their realisation and creating custom annotations. We observed a relatively high number of unclear cases that would require an elaborate phonetic analysis in a further work. The difficulty in discriminating between different phonetic units is due not only to the type of segments (which are often difficult to distinguish), but also to the fact that individual words cannot be heard in a loop, since the corpus is not word but turn-aligned.

#### About the dialect use: Ikavian and (Ij)ekavian variants

Since the siblings were one of the few participants who reported to have spoken a dialect (Ikavian), we additionally analysed their use of dialect features in conversations in which they took the role of giving indications. We focused on detecting whether their use of Ikavian *jat* reflexes was consistent throughout the transcript, and whether there are differences between the two in using them. For finding Ikavian words we first selected all normalised words (in transcript view: *Annotations/Non-standard/spoken*) and then annotated the Ikavian words amongst them by adding custom annotations (in transcript view: *Annotate*). We also annotated their non-Ikavian counterparts as well as other non-Ikavian variants whenever they were present in the transcript with a different tag. Then, we exported the annotations and created a comparison table (Table 4).^48^

**Table 4:** Frequency of use of words with Ikavian (i) and (Ij)ekavian (ije/je/e) *jat* reflex by Marija (map task: GK_01_B) and Darko (map task: GK_04_A)

English translation	Lemma	Marija	Darko
*where*	d**i**	2	1
	gd**je**	1	1
*up*	gor**i**	9	0
	gor**e**	5	9
*down*	dol**i**	4	0
	do**lje**	1	0
	dol**e**	0	5
*somewhere*	negd**i**	1	1
	negd**je**	0	0
*left*	l**i**vo	0	0
	l**ije**vo	9	8
*total*		32	25
*total Ikavian*		16	2

Croatian standard variants of the words in table are: *gdje, gore, dolje, negdje,* and *lijevo*. In one Darko’s segment that was transcribed as *gdje* it was unclear whether *di* or *gdje* was used. We did not consider that instance in this table

As it can be observed in Table 4, both siblings used not only Ikavian, but also (Ij)ekavian variants in map task conversations. In comparison, an Ikavian participant originating from Croatia (Ante*) was consistent in using only Ikavian features throughout the transcript. Possibly, the siblings used Ijekavian features next to Ikavian because their mother originates from Žepče, where Ijekavian is spoken,^49^ and they might already use the “mixed” variety at home.^50^ However, it could also be the case that they tried to avoid exclusively Ikavian features because this variety is not encoded in any BCMS standard language and it has low prestige in Bosnia and Herzegovina, in contrast to Ijekavian.^51^ For that reason, speakers might have adapted to their non-Ikavian interlocutors by avoiding the forms that are marked dialectally or geographically (see *levelling* in Aalberse & Muysken, 2013, pp. 8–9). The use of different *jat* reflexes could also be due to the difference of treatment of long and short *jat* reflexes: the long *jat* reflex in *lijevo* is consistently realised as *ije*, while reflexes in other words that contain short *jat* are less consistent.^52^ Other examples of mixing variants of *jat* vowel reflex by a BCMS heritage speaker have been observed by Hansen et al., (2013, p. 24), and would be an interesting subject for further research of dialect levelling.

#### Summary

In this section we illustrated how the corpus can be used on the example of an investigation of similarities and differences that the siblings Marija and Darko show regarding lexical, morphosyntactic and phonetic peculiarities, as well as dialectal variants. We found no major differences between the two siblings regarding the features we investigated. The siblings exhibited great difficulties in lexical retrieval, showed the same type of deviation in realisation of the phonetic segment** /**dʑ/, and they both mixed dialectal variants with standard language *jat* reflex variants. Darko, the younger sibling, additionally showed morphosyntactic deviations from the standard norm that were absent in Marija’s utterances. However, since Marija did not use the same constructions in her indications (with *dok* ‘until’), this difference might just be due to chance. While this comparison is only of exemplary nature, and an overall assessment of heritage language proficiency would require analysis of more features and more data, we assess that in our case study no sufficient evidence was found that Marija, the older sibling, is more proficient in BCMS than her younger brother. Possibly, this is due to the small age difference of only 2 years.

While analysing the data, the corpus platform showed to be most useful for lexical searches, searches of pre-annotated data (non-BCMS segments, normalised segments, etc.), as well as for creating and sharing custom annotations. The latter was particularly helpful for categorising phonetic peculiarities among multiple raters. The possibility to access detailed metadata about speakers also proved relevant for the interpretation of the findings regarding phonetic and dialectal variants. The corpus platform showed to be useful for enabling access to pre-annotated (non-BCMS) and structured data that can be further elaborated. However, like in any, especially small corpus, pre-annotated as well as custom observations regarding the quantitative distribution of linguistic features should be treated with caution. For instance, while some speakers, like Marija, do not show any recurrent deviations in verbal aspect, it does not necessarily mean that they have an excellent command of it, but that they either didn’t had to use particular structures in their conversation, or that they used other structures (or avoiding strategies) instead. Similarly, speakers that used (Swiss-)German words in their map tasks are not necessarily less proficient in BCMS lexicon, but they possibly choose to deliberately use (Swiss-)German words as a strategy for more fluent speech.

## Discussion

The BCMS map task corpus offers interactive and collaborative work on the corpus, and a possibility to download different versions of the transcripts, metadata and (pre-)annotated data on local machines, where users can profit from other software and processing options. As such, this corpus represents a compromise between web-(only)-based environments, advocated by Kemps-Snijders et al. (2008), and the “download first, then process” paradigm. Regarding the possibility of accessing corpus data after querying quantitative information, the metadata filter in this corpus is comparable to those of spoken language tools such as Lexical Explorer^53^ and ZuMal.^54^ Being able to explore quantitative data for each transcript at a glance helps users to choose transcripts to examine, and it can point out speakers’ individual tendencies regarding particular linguistic and paralinguistic features. Further work on the transcripts is facilitated by the possibility to add, export and share custom annotations.

While the map task corpus platform is created for one specific corpus, its modular structure is adaptable to other resources that have the same transcript or metadata structure. Another advantage of this corpus platform is its simplicity, which is the “key issue in spreading corpus use in and beyond the research community” (von Waldenfels & Woźniak, 2017, p. 156). As observed by Fandrych et al. (2016), working with corpora of spoken language in an online setting is often challenging for students, researchers, and teachers. Since typical users of spoken language corpora have high expectations regarding platform usability, but they do not want to invest much time into learning to use new software or techniques (ibid.), we implemented simple querying and annotation techniques, which demand no additional training.

The case study showed how the corpus interface can be used for a linguistic study of heritage BCMS. The pre-selection of metadata, as well as the possibility to work upon pre-annotated features proved to be very useful for the aims of our investigation, especially regarding analysis of lexical peculiarities. The most challenging task was the annotation of phonetical deviations. This is mostly due to the fact that the corpus is turn-aligned, while it should ideally be word-aligned, in order to permit repetitive hearing of the same word (or multi-word segment).

Needless to say, the semi-orthographic transcription used in this corpus reflects transcribers’ subjective interpretations that might differ from the interpretations of other users. Since the transcripts are short, it might also lead to a slight over- or underrepresentation of features such as number of pauses and elongations in corpus counts section. However, we consider pronunciation-based transcription and the annotation of non-vocal segments and pauses a very good compromise between readability of the transcript and faithfulness to the original source.

Another limitation of the corpus is given by the type of data: while map tasks are well adapted for pilot studies, users must bear in mind that they represent an experimental situation which is (deliberately) thematically narrow. Besides, map task conversations contain for the most part encodings of directions that are embedded in a situation which is likely to lead to unusual collocations such as “ideš do büroklammera” (‘you go towards the paperclip’). Despite these challenges, the map task corpus of heritage BCMS represents a valuable insight not only into the language contact between the heritage and the majority language, but also between different BCMS varieties among themselves. The corpus can be used, for instance, as a resource for studying phonetic peculiarities found in other Slavic heritage languages, such as distinguishing contrasting pairs like palatalised and non-palatalised consonants (Sussex, 1993, p. 1014), aspiration of initial voiceless stops (see Brehmer & Kurbangulova, 2017), and the replacement of Slavic [ł] by a “less velar” or “clearer” [l] (see Sussex, 1993, p. 1013; Recasens & Espinosa, 2005). It can also be used for investigating disfluency patterns (Yılmaz & Özsoy, 2020), and discourse markers used by heritage speakers (see Hlavac, 2006), as well as for studying language accommodation to other speakers in general, and to speakers of other BCMS varieties in particular (Giles & Ogay, 2007; Ljubešić et al., 2019). Teachers and students of BCMS in the diaspora can profit from the text-audio alignment, and the possibility to annotate the transcripts and share their results with their peers. The pre-selection of transcripts according to metadata such as speaker’s origin or the proportion of normalised tokens can help teachers choose appropriate transcripts for didactic use in the classroom.

In future work, we aim to further develop the custom annotation functionality, perform word-alignment by using forced alignment techniques, and include a quality score based on a measure of overlap between original and drawn route. We also aim to use the technologies and workflows developed for this corpus platform to enhance the work on corpora of heritage Albanian we are currently working on.

## Appendix 1: Map task images (stimuli)

**Table:**

Map task	croatian	bosnian	serbian	German	Swiss–German	English
A	tržnica	pijaca	pijaca/pijac	Markt	Markt	market
A	(štipaljka za vrećice)	(štipaljka za kese/vrećice)	(štipaljka za kese)	Verschlussklemme	Klammere	bag clip
A	cikla	cvekla/cikla	cvekla	Rande	Rande	beetroot
A	šnala/kopča	šnala/kopča	šnala	Haarspange	Haarspängeli	hair clipper
A	heftalica	heftalica	heftalica	Hefter	Bostitsch	stapler
A	čvor	čvor	čvor	Knoten	Chnopf	node
A	ljestve	ljestve	merdevine/lestve	Leiter	Laitere	ladder
A	smeće (odlagalište)	smeće (odlagalište)	smeće/đubre (deponija)	Abfall	Abfall	garbage
A	ključanica	ključanica/ključaonica	ključaonica	Schlüsselloch	Schlüsselloch	key hole
A	kutlača	kutlača	kutlača	Suppenlöffel	Suppelöffel	soup spoon
A	šišmiš	šišmiš	slepi miš	Fledermaus	Fledermuus	bat
B	škare	makaze	makaze	Schere	Schär	scissors
B	ljenjivac	ljenjivac	lenjivac	Faultier	Fuultier	sloth
B	gradilište	gradilište	gradilište	Baustelle	Baustell	construction site
B	zdjela	zdjela/činija	činija/zdela	Schüssel	Schüssle	bowl
B	kruh	hljeb/kruh	hleb	Brot	Brot	bread
B	cvjetača	karfiol/cvjetača	karfiol	Blumenkohl	Bluemechool	cauliflower
B	spajalica	spajalica	spajalica	Büroklammer	Bürochlammere	paperclip
B	šipak	šipak/šipurak	šipak	Hagebutte	Hagebutte	rose hip
B	odvijač	odvijač / šrafciger	šrafciger	Schraubenzieher	Schruubezieher	screwdriver
B	vjetrobran	vjetrobran	vetrobran/šoferšajbna	Windschutzscheibe	Windschutzschiibe	windshield
B	puran/purica	ćuran/ćurka	ćuran/ćurka	Truthahn	Truthahn	turkey

## Appendix 2: Participants^55^

**Table:**

Session number	Speaker ID	Pseudonym	Gender	Interlocutor (Pseudonym)	“Which language did you speak in the map task?”	“I know my interlocutor well” 1: disagree; 5: agree
1	DL_01	Snežana	F	Ante	Serbian	5
	DL_02	Ante	M	Snežana	Croatian	5
2	DM_01	Matea	F	Iskra	Croatian	5
	DM_02	Iskra	F	Matea	Serbian	5
3	GK_01	Dragan	M	Marija	Serbian	1
	GK_02	Marija	F	Dragan	Croatian	1
4	GK_03	Ljerka	F	Lana	German–Croatian	5
	GK_04	Lana	F	Ljerka	Serbian/Bosnian/German	5
5	GK_05	Ivan	M	Dejan	Croatian	2
	GK_06	Dejan	M	Ivan	Bosnian	1
6	GK_07	Darko	M	Marina	Croatian	4
	GK_08	Marina	F	Darko	Croatian	5
7	HK_01	Stanko	M	Tanja	Serbian	1
	HK_02	Tanja	F	Stanko	Serbian	1
8	JA_01	Dora	F	Renata	Serbian	1
	JA_02	Renata	F	Dora	Croatian	1
9	JR_01	Gordana	F	Antonia	N.A. (Serbian)	1
	JR_02	Antonia	F	Gordana	Croatian	1
10	LM_01	Ana	F	Mila	Serbian	2
	LM_02	Mila	F	Ana	Serbian	2
11	LM_03	Dijana	F	Tina	Serbian	2
	LM_04	Tina	F	Dijana	Croatian	1
12	MB_01	Davor	M	Suad	Bosnian	5
	MB_02	Suad	M	Davor	Bosnian	5
13	OW_01	Anel	M	Iva	Bosnian	2
	OW_02	Iva	F	Anel	Croatian and Swiss–German	1
14	SB_01	Iva	F	Suzana	Croatian	2
	SB_02	Suzana	F	Iva	Serbian	2
15	SV_01	Nemanja	M	Zvonimir	Serbian	2
	SV_02	Zvonimir	M	Nemanja	Croatian	4

## Footnotes

1 In this paper we use the definition of heritage languages by Rothman (2009, p. 156): “A language qualifies as a heritage language if it is a language spoken at home or otherwise readily available to young children, and crucially this language is not a dominant language of the larger (national) society”.

2 BFS, *Die üblicherweise zu Hause gesprochenen Sprachen, 2017–2019, kumuliert:* https://www.bfs.admin.ch/bfs/de/home/statistiken/bevoelkerung/sprachen-religionen/sprachen.assetdetail.16404820.html (08.07.2022). Results are given for ‘Serbian-Croatian’ (“Serbisch-Kroatisch”). Bosnian and Montenegrin are not included.

3 The Štokavian dialect (named after the word for *what* ‘što’) embraces the main territory of the South Slavic dialectal continuum, namely Bosnia and Herzegovina, Montenegro and most part of Croatia and Serbia. The Štokavian dialect was (unofficially) chosen to be the basis for a joint standard language in mid nineteenth century in a meeting known as Vienna Literary Agreement (see Kapović, 2010, p. 128). In this paper we use the term *dialect* for non-standard varieties and *language* for standard varieties. Since Štokavian is at core of standard BCMS languages, these two denominations may overlap.

4 Chiba University Japanese Map Task Dialogue Corpus (MapTask): http://research.nii.ac.jp/src/en/MapTask.html (08.07.2022).

5 HZSK. 2010. “HAMATAC—the Hamburg MapTask Corpus.” Archived in Hamburger Zentrum für Sprachkorpora. Version 0.3. Publication date 2010–09-16. http://hdl.handle.net/11022/0000-0000-6330-A (08.07.2022).

6 BeMaTaC: https://www.linguistik.hu-berlin.de/de/institut/professuren/korpuslinguistik/forschung/bematac (08.07.2022).

7 Montclair Map Task Corpus: https://digitalcommons.montclair.edu/mmt_corpus/ (08.07.2022).

8 See “Appendix 1” for the list of stimuli. In the map task A, we selected a word that to our knowledge is still not codified in standard BCMS-varieties (*bag clip*), in order to see how speakers will describe it to their interlocutors.

9 Since all participants signed a declaration of consent, and since their names are pseudonymised, we did not ask for Institutional Review Board approval. Instead, the declaration of consent was reviewed by a legal practitioner specialised in data protection. In addition, we ensured that corpus users agree to our Terms and Conditions, which state that the use of corpus data is permitted only for research, teaching or study purposes.

10 The token count reported above includes also the occasional comments by the instructors. If only the conversations of the participants are regarded, the corpus contains 12,797 tokens.

11 See “Appendix 2” for the list of participants.

12 We provide the number of given answers in parentheses whenever not all participants provided this information.

13 For one speaker (pseudonym: *Dijana*) we have no information about father’s origins.

14 Other participants either did not specified their ethnic background or they came from ethnically mixed families.

15 One participant wrote that her first language is Serbo-Croatian, but when asked which variety she used in the map task, she wrote “Officially: Bosnian. My opinion: Serbian Ijekavian with German vocabulary”. She also added a comment in which she explains her struggles in more detail: “I think BCMS is a beautiful language, but it has always been difficult for me to position myself. That's why I never went to a ‘Yugo-school’ [courses of BCMS offered for children living abroad]. Should I have taken the Serbian or the Bosnian?”.

16 The Common Slavonic *jat* vowel (*ě) has three reflexes in Western South Slavic languages: *ije/je* (Ijekavian), *e* (Ekavian), and *i* (Ikavian), as in *mlijeko*, *mleko* and *mliko* (‘milk’). In the questionnaire we referred to the Ikavian/Ijekavian/Ekavian *jat* vowel reflex as *pronunciations* (German: *Aussprache*) to make the participants understand what is meant by it without using the linguistic terminology.

17 FOLKER: https://exmaralda.org/de/folker-de/ (08.07.2022).

18 OrthoNormal: https://exmaralda.org/de/offizielle-version/ (08.07.2022).

19 TEI Guidelines Version 4.2.1 (Transcriptions of Speech). Available at: https://tei-c.org/release/doc/tei-p5-doc/en/html/TS.html (08.07.2022).

20 Classla 1.0.0 (CLASSLA Fork of Stanza for Processing Slovenian, Croatian, Serbian, Macedonian and Bulgarian). Available at: https://pypi.org/project/classla/ (08.07.2022).

21 Universal POS tags: https://universaldependencies.org/u/pos/.

22 Serbo-Croatian MULTEXT-East Specifications. Available at: http://nl.ijs.si/ME/V6/msd/html/msd-hbs.html (08.07.2022).

23 We omitted non-BCMS words, hesitation and acknowledgment tokens in the tagging process.

24 DataTables: https://datatables.net/ (08.07.2022).

25 Since most participants were born in Switzerland, we choose the father’s place of birth as an approximation representing the place of origin in former Yugoslavia.

26 For instance, depending on the question type, we made responses lowercase/uppercase, we corrected typing errors, and we added a country code, longitude, and latitude for each location.

27 For calculating the type/token ratio we used the moving average type/token ratio (MATTR, Covington & McFall, 2010) with the window size of 100.

28 The relative counts were calculated by dividing the number of occurrences of a particular phenomenon with the total number of tokens (multiplied with 100). There are however three participants who utter less than 100 words, which might distort the comparisons among counts. These speakers can be filtered out with the functionality “Filter speakers with > 100 tokens”.

29 Corpus of Serbian Forms of Address (demo version). Accessible at https://gitlab.uzh.ch/uzh-slavic-corpora/serbian-forms-of-address (05.09.2022).

30 Praat (Boersma, 2001, www.fon.hum.uva.nl/praat/) is a tool for phonetic analysis.

31 Custom tags can contain letters (A-Za-z), digits (0–9), hyphens (-), underscores (_), and be up to 50 characters long.

32 MongoDB: https://www.mongodb.com/ (08.07.2022).

33 Flask: https://palletsprojects.com/p/flask/ (08.07.2022).

34 ZuCoSlaV: Zurich Corpora of Slavic Varieties. Available at: https://gitlab.uzh.ch/uzh-slavic-corpora (08.07.2022). The link to the map task demo page is provided at https://gitlab.uzh.ch/uzh-slavic-corpora/BCMS-map-tasks (05.09.2022).

35 Licence details are available at: https://creativecommons.org/licenses/by-nc-sa/4.0/ (08.07.2022).

36 It must be mentioned that the questions in the two surveys were not identical: in Romić’s (2016) questionnaire it was specified that the other people from former Yugoslavia are one's friends, and in Mayer & Lemmenmeier-Batinić's questionnaire the question only mentioned “people from former Yugoslavia”. In addition, Romić's questionnaire contained predefined answers (“I speak German with my siblings”/ “I speak BCS with my siblings”, etc.), whereas Mayer & Lemmenmeier-Batinić's questionnaire offered answers with a 5-point Likert scale, representing the values from “never” to “always”. Note that in Mayer & Lemmenmeier-Batinić's survey many more participants reported to *always* speak BCMS with either their siblings or with people form former Yugoslavia than in Romić's survey.

37 For BCMS affricates we use the phonetic notation and denomination proposed by Horga & Liker (2016, p. 266).

38 The effect the interlocutor might have on their partner’s speaking performance has been subject to scrutiny (see Foot, 1999). Regarding the familiarity with the interlocutor, O’Sullivan (2002) and Norton (2005) reported that knowing the interlocutor may positively affect speaker’s performance. However, these studies have not been conclusive (see Son, 2016, p. 46).

39 To compare the language profiles of the two sibings, we searched for the two speakers in the column *Pseudonym* in the metadata table with the regular expression *Marija*|*Darko.* We exported the results, transposed the table, and selected the relevant fields regarding the language profile shown in Table 2, and quantitative information shown in Table 3. In answers that refer to a 5-point Likert scale, “1” stands for “disagree/false”, and 5 stands for “agree/correct”.

40 The question about media consumption was originally given for each BCMS-country separately. The score represented in Table 2 is the highest score the participants gave regarding their media consumption related to any of the four countries.

41 We regarded a word as correct if it was semantically correct, even if it showed phonetic or morphosyntactic deviations from the standard. For instance, *smeče* [smeʧe] was marked as correct although the standard term is *smeće*/smetɕe/(‘garbage’). We reduced the set to 10 words instead of 11 by disregarding the word for *construction site* (which was almost always produces as the germanism *bauštela* ‘Baustelle’ in map task B), and for *bag clip* (map task A).

42 Non-BCMS tokens can be shown with the help of the functionality *Annotations/Non BCMS.*

43 For reasons of simplicity, glossing conventions (Leipzig Glossing Rules) are used only in those examples in which the authors consider them necessary to understand the example.

44 Excerpts are shown so as they were transcribed, without adjusting them to the BCMS orthographic rules.

45 We asked three independent raters who differentiate between /dʒ/ and /dʑ/ in perception and production to annotate all instances of *đ* in the transcripts (17 for Marija and 4 for Darko) and categorise them [dʑ], [dʒ] or *unclear.* All raters perceived [dʒ] in Darko’s and Marija’s turns: there were at least 8, and at most 10 [dʒ] segments per rater. In Darko’s turns three out of four segments were categorised as [dʒ] by all raters. There was less agreement for Marija’s segments, in which only two segments were categorised as [dʒ] by all raters. Overall, the raters reached a fair agreement (Fleiss’ kappa 0.25).

46 The use of /tɕ/ (ć) instead of /ʧ/ (č), and */*dʑ/ (đ) instead of */*dʒ/ (dž) is frequently encountered within Muslim population and in urban centres of central Bosnia and Hercegovina (see Peco, 2000). In western Herzegovina, where siblings’ father originates, the difference between the affricates is well preserved (see Peco, 2000, p. 214). Regarding the current situation in Žepče, where their mother originates, we only found data representing the Muslim population, which suggests that *dž* is realised as [dʑ] (đ), at least what the word *džamija* (‘mosque’) is concerned, see Halilović et al., (2020, pp. 17, 210).

47 For instance [dʒ] is also evidenced in *dodžeš* [dodʒeʃ] ’you come’ (transcript: DM_01_2), *prodžeš* [prodʒeʃ] ‘you go by’ (transcript: JA_01_B), and the unvoicing of /dʑ/ to /tɕ/ is evidenced in *izmeću* [izmetɕu] ‘between’ (transcript: SB_01_B).

48 We shared the annotations of *jat* reflexes used for this section on the Uploads page (*MT_Paper_Darko_Ikavian* and *MT_Paper_Marija_Ikavian*).

49 It has to be mentioned that Žepče is situated close to the Ijekavian and Ikavian isogloss, and mixtures of Ikavian and Ijekavian have been evidenced in this area (Rešetar, 1907,p. 65; Baotić, 2012, p. 157).

50 Another possibility is that the Ikavian *jat* vowel reflex in New Štokavian Ikavian dialect (‘novoštokavski ikavski dijalekt’) is often inconsistent in the first place (see Lisac, 2003, p. 174), but this does not explain the variation in the use of Ikavian variants within speakers that originate from the same place.

51 The metadata do not contain information about which attitudes these speakers have towards Ikavian.

52 There is a preference for Ikavian variants in Marija’s utterances, who utters them more frequently in four out of five lemmas (*di, gori, doli, negdi*). Darko, on the other hand, shows a preference for Ekavian variants regarding the lemmas *gore* and *dole.* It's interesting that all three variants for ‘up’ (*doli, dolje, dole*) are encountered, although Darko uses exclusively *dole*, and Marija *doli* and *dolje.*

53 Lexical Explorer: https://www.owid.de/lexex/ (14.10.2022).

54 ZuMal is one of the corpus access prototypes developed in the ZuMult project: http://zumult.ids-mannheim.de/ProtoZumult/index.jsp (08.07.2022).
